# Rapid evolutionary turnover underlies conserved lncRNA–genome interactions

**DOI:** 10.1101/gad.272187.115

**Published:** 2016-01-15

**Authors:** Jeffrey J. Quinn, Qiangfeng C. Zhang, Plamen Georgiev, Ibrahim A. Ilik, Asifa Akhtar, Howard Y. Chang

**Affiliations:** 1 Center for Personal Dynamic Regulomes, Stanford University School of Medicine, Stanford, California 94305, USA;; 2 Department of Bioengineering, Stanford University School of Medicine and School of Engineering, Stanford, California 94305, USA;; 3 Max Planck Institute of Immunobiology and Epigenetics, 79108 Freiburg im Breisgau, Germany

**Keywords:** roX, lncRNAs, dosage compensation, RNA structure, ChIRP, *Drosophila*

## Abstract

Many long noncoding RNAs (lncRNAs) can regulate chromatin states, but the evolutionary origin and dynamics driving lncRNA–genome interactions are unclear. We adapted an integrative strategy that identifies lncRNA orthologs in different species despite limited sequence similarity, which is applicable to mammalian and insect lncRNAs. Analysis of the roX lncRNAs, which are essential for dosage compensation of the single X chromosome in *Drosophila* males, revealed 47 new roX orthologs in diverse Drosophilid species across ∼40 million years of evolution. Genetic rescue by roX orthologs and engineered synthetic lncRNAs showed that altering the number of focal, repetitive RNA structures determines roX ortholog function. Genomic occupancy maps of roX RNAs in four species revealed conserved targeting of X chromosome neighborhoods but rapid turnover of individual binding sites. Many new roX-binding sites evolved from DNA encoding a pre-existing RNA splicing signal, effectively linking dosage compensation to transcribed genes. Thus, dynamic change in lncRNAs and their genomic targets underlies conserved and essential lncRNA–genome interactions.

## Abstract (precis)

In this study, Quinn et. al. used an integrative strategy based on matching focal and repeated RNA secondary structures and other RNA features that uncovers novel lncRNA orthologs despite limited sequence similarity. This method was applied to *Drosophilia* roX1 and roX2 RNAs, and 47 new roX RNAs across ∼40 million years of evolution were discovered.

Eukaryotic genomes are replete with long noncoding RNA (lncRNA) genes that are diverse, tightly regulated, and engaged in numerous biological processes (Ponting et al. 2009; Cabili et al. 2011; Derrien et al. 2012; Rinn and Chang 2012). LncRNAs differ from protein-coding genes in many ways (Quinn and Chang 2016) and, in particular, are often less conserved at the level of primary sequence (Chodroff et al. 2010; Ulitsky et al. 2011). This is because many of the selection pressures that constrain protein-coding primary sequences do not apply to lncRNAs, such as maintenance of ORFs and codon synonymy. The low primary sequence conservation has led some to dismiss lncRNAs as transcriptional noise (Babak et al. 2005; van Bakel et al. 2010) and also hinders the discovery of lncRNA orthologs in other genomes by sequence homology. These issues in turn limit the investigation of lncRNAs’ evolutionary origins and dynamics, conserved elements, and functions. Examples of such evolutionary analyses are scarce yet valuable, such as evidence for the independent evolutionary origins of the mammalian dosage compensation lncRNAs Xist and Rsx, with Xist having arisen from a pseudogenized protein-coding gene (Duret et al. 2006; Grant et al. 2012). Besides their primary sequence, other lncRNA features are often conserved, including syntenic relationships to other genes (i.e., neighboring genes), short sequence homology (referred to here as “microhomology”), and secondary structure (Chodroff et al. 2010; Ulitsky et al. 2011; Bussotti et al. 2013; Hezroni et al. 2015). Despite many predictions from RNA sequencing (RNA-seq) data (Necsulea et al. 2014; Hezroni et al. 2015), few lncRNA orthologs that function across species have been experimentally verified.

Although a growing list of lncRNAs is known to interact with the genome (Mondal et al. 2010), little is known about how these interactions evolve or what features are conserved. ChIRP (chromatin isolation by RNA purification) and related technologies have proven useful for mapping and studying the genomic binding sites of such chromatin-associated lncRNAs, such as Xist and roX lncRNAs (Chu et al. 2011, 2015; Simon et al. 2011). However, no comparative genomic analyses have yet been done to study the pattern of lncRNA occupancy in different species. Comparative genomic analyses of transcription factor-binding sites and enhancers have revealed the ways evolution has shaped these functional genomic elements (He et al. 2011; Villar et al. 2015). Genomic occupancy maps of lncRNA orthologs in several species, ideally in vivo, may reveal the evolutionary forces shaping lncRNA–genome interactions.

An ideal model system for studying the evolution of lncRNA–genome interactions is the dosage compensation system in *Drosophila melanogaster* because it employs two lncRNAs (roX1 and roX2) that are essential for dosage compensation and bind to hundreds of distinct sites on the X chromosome. Dosage compensation is the epigenetic phenomenon by which gene expression from the single X chromosome in males is doubled to match gene expression of females’ two X chromosomes. The roX lncRNAs are critical for assembling, targeting, and spreading the dosage compensation complex (DCC; a chromatin-modifying complex) along the X chromosome to high-affinity sites (HASs) (Alekseyenko et al. 2008; Chu et al. 2011; Conrad and Akhtar 2011). Genetic ablation of *roX* genes or any of five DCC proteins results in failed dosage compensation and male-specific lethality (Meller and Rattner 2002). Despite the fact that roX1 and roX2 are functionally redundant, they differ greatly in sequence and size (3.7 kb and 0.6 kb, respectively). The functional redundancy between roX RNAs is primarily attributed to a short, repeated sequence motif (the 8-nucleotide roXbox motif) embedded in stem–loop structures in both roX1 and roX2 (Park et al. 2008; Ilik et al. 2013). Previous roX ortholog search efforts identified eight roX1 and nine roX2 orthologs in other species using whole-gene BLAST (Park et al. 2007; Alekseyenko et al. 2013) or structure detection by sequence covariation (Fig. 1B; Byron et al. 2010). However, these strategies failed to identify roX orthologs in many *Drosophila* species, as the primary sequence identity between discovered orthologs was close to random (Park et al. 2007). Nonetheless, these studies highlighted evolutionarily conserved structures that are essential to roX function (Park et al. 2008).

**Figure 1.**
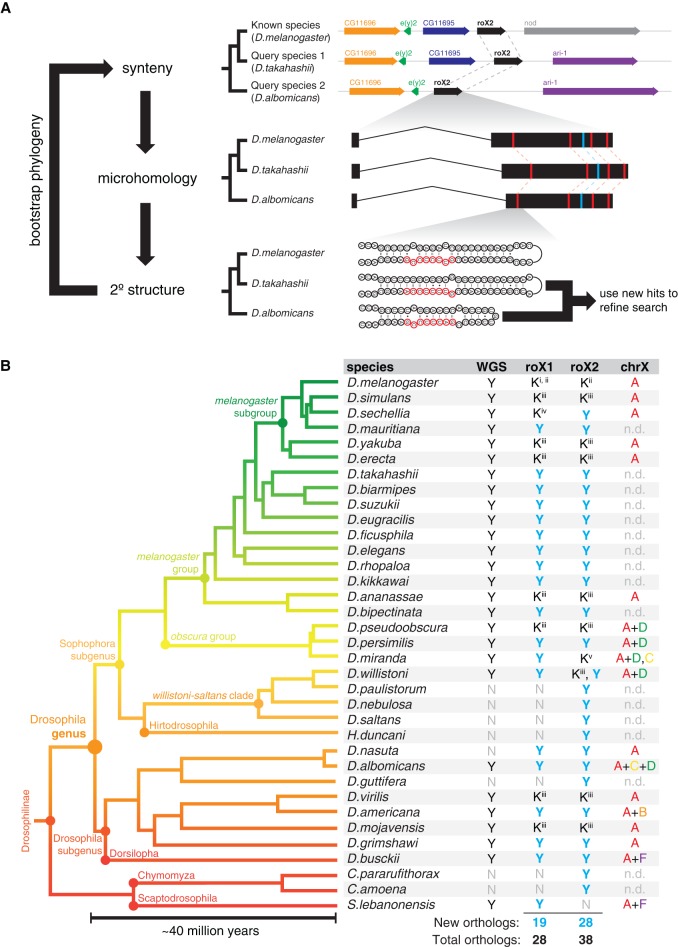
Summary of lncRNA ortholog search strategy and queried species. (*A*) The search strategy found lncRNA orthologs in query species by integrating synteny, microhomology, and secondary structure features of a known lncRNA. The search features were iteratively refined by bootstrapping new ortholog candidates and the phylogenetic relationships between queried species. To initiate the search, a priori knowledge of the lncRNA in only a single species is needed. (*B*) Phylogenetic tree of the 35 Drosophilid species queried in this study. Whole-genome sequencing (WGS) assemblies were available for 27 species. Nine roX1 and 10 roX2 orthologs have previously been described ([K] known roX ortholog from Amrein and Axel 1997 [i]; Meller et al. 1997 [ii]; Park et al. 2007 [iii]; Byron et al. 2010 [iv]; Alekseyenko et al. 2013 [v]); our search identified 47 new roX orthologs. (Y) New ortholog; (N) no ortholog found. X chromosome karyotypes are indicated by Müller elements. (n.d.) No data.

Here, we describe a lncRNA ortholog search strategy that integrates synteny, microhomology, and secondary structure (Fig. 1A). Using this strategy, we discovered 47 previously undescribed roX orthologs in 35 diverse fruit fly species. We compared these roX orthologs and mapped the genome-wide binding sites of roX lncRNA orthologs in four species and discovered evolutionary principles that determine lncRNA structure, function, and genomic binding sites.

## Results

### Identification of 47 new roX lncRNA orthologs

Our lncRNA ortholog search strategy is parameterized on three heuristics (synteny, microhomology, and secondary structure) and iteratively bootstraps new ortholog hits and the phylogenetic relationships between query species (Fig. 1A; see the Materials and Methods for a detailed description). First, we searched for synteny blocks likely containing the *roX1* or *roX2* loci, employing a computational or analog method (tBLASTn or degenerate PCR, respectively) depending on the availability of completed genome assemblies for the subject species. Next, we homed in on roX orthologs by searching for incidences of microhomology (roXbox motifs) and structure (roXbox stem–loops) within the identified synteny window, which thus served as landmarks for the roX ortholog candidates. Last, we leveraged new lncRNA ortholog hits and the phylogenetic relationships between query species to iteratively refine the search parameters. For example, we collapsed roXboxes from each newly identified roX ortholog to improve the motif; we also searched synteny windows matched to that of its closest relative's *roX* locus. In this way, the search strategy became more powerful with each new ortholog identified. This bootstrapping strategy differs from prior approaches based entirely on synteny (Ulitsky et al. 2011) and proved essential for lncRNA ortholog discovery.

The *Drosophila* genus is highly diverse, comprising nearly 2000 named species with well-characterized phylogenetic relations (Fig. 1B; van der Linde et al. 2010) that diverged ∼40 million years ago, as defined by the *Sophophora*–*Drosophila* subgenera divergence (Russo et al. 1995; Robe et al. 2010). To put this evolutionary distance in perspective, humans and spider monkeys also diverged ∼40 million years ago, as did dogs and bears, although Drosophilids have undergone orders of magnitude more generations during this time. In this study, we selected 27 species with sequenced genomes plus eight additional species to maximize phylogenetic diversity, including the outgroup genera *Chymomyza* and *Scaptodrosophila* (Fig. 1B).

**Figure 2.**
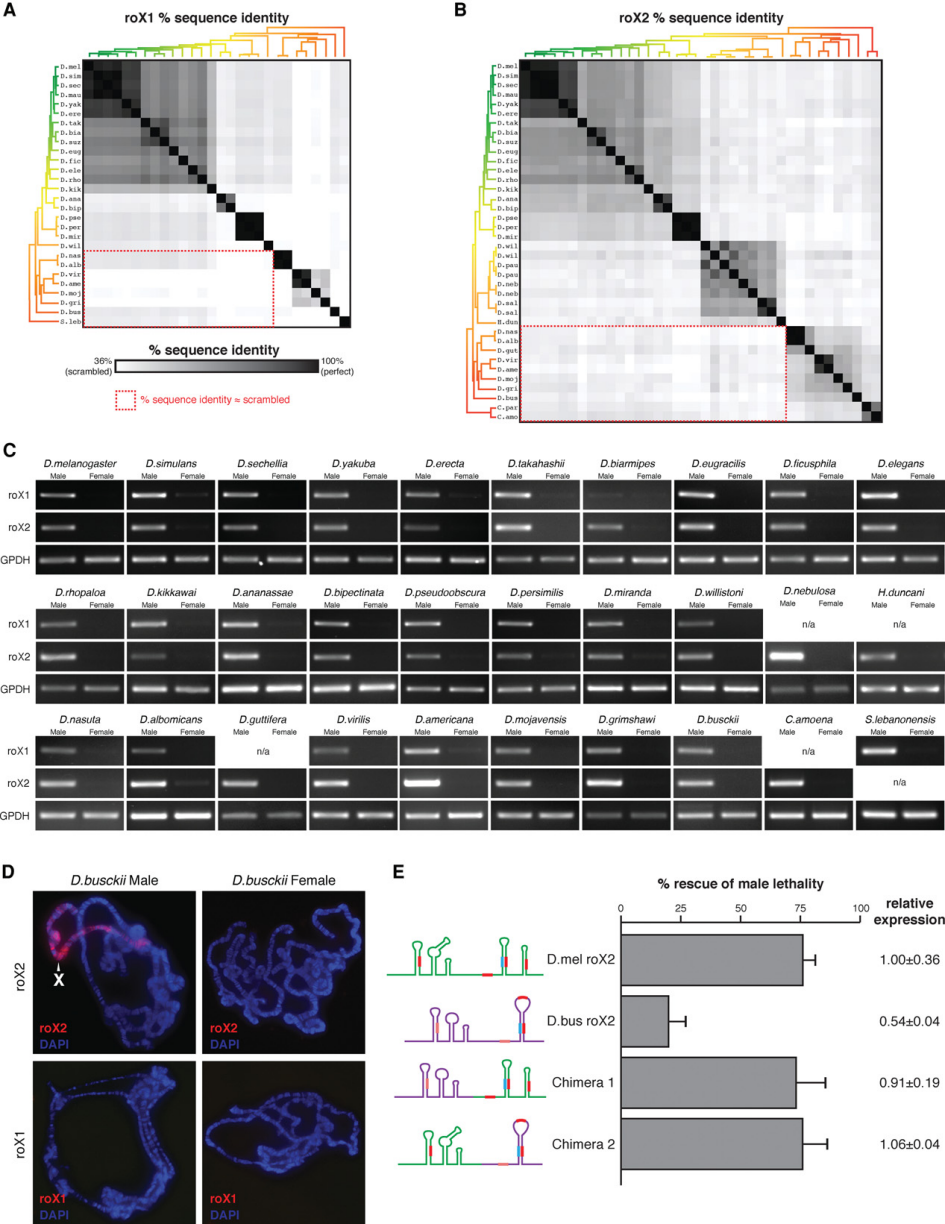
Identified roX candidates are bona fide orthologs despite sequence divergence. (*A*,*B*) Heat map showing the sequence conservation between the identified roX1 (*A*) and roX2 (*B*) ortholog candidates relative to the lower limit of homology (scrambled sequences, 36%). Phylogenetic trees as in Figure 1B. Red dashed boxes highlight exceptionally poor conservation between distantly related species. (*C*) RT–PCR of roX1, roX2, and GPDH RNA in male and female flies. roX1 and roX2 orthologs exhibit strong male-biased expression; GPDH mRNA is a sex-independent control. (n/a) No ortholog found. (*D*) RNA FISH of roX1 and roX2 in polytene chromosomes from male and female *Drosophila busckii* larvae. roX2 paints the male X chromosome (white arrowhead) but not the female X; roX1 was not detected. (*E*) Rescue of male lethality in *roX*-null *D. melanogaster* (D.mel) males by transgenic *D. busckii* (D.bus) roX2 or chimeric *busckii–melanogaster* roX2. RNA cartoons depict secondary structures, with roXboxes (red) and inverted roXboxes (blue) indicated. Error bars show standard deviation. Expression was calculated relative to wild-type roX2 transgene ± standard deviation.

Our search found 47 new roX ortholog candidates (19 roX1s and 28 roX2s) in addition to those previously described, more than tripling the number of known roX orthologs (66 total) (Fig. 1B). In the few cases where roX orthologs could not be identified, a complete genomic assembly was lacking or incomplete or there was syntenic disruption at the *roX* locus. Curiously, the search identified three high-scoring roX homolog candidates in *Drosophila willistoni*; close analysis of these candidates in *D. willistoni* and its relatives indicated that *roX2* was duplicated in the *willistoni–saltans* clade after the divergence of *Hirtodrosophila duncani*, resulting in up to three functional *roX* genes (we call this *roX2* paralog “*roX3*”) (Supplemental Fig. S1). The roX orthologs identified exhibit exceptionally low primary sequence conservation, dropping to the lower limit of homology (i.e., indistinguishable from scrambled sequences) when comparing sequences between *Sophophora* and *Drosophila* subgenera or outgroups by multiple sequence alignment (Fig. 2A,B). The bootstrapping approach was critical because the neighboring genes at the *roX* loci differed between species, such as the *roX2–nod* synteny block in the *melanogaster* subgroup versus *roX2–ari-1* in nearly all other species (Supplemental Fig. S2).

Additionally, to test the generalizability of this search strategy, we searched for orthologs of HOTAIR lncRNA in 43 vertebrate genomes, initiating the search with only the sequence of human HOTAIR in the *HOXC* cluster. We identified the orthologous *HOTAIR* locus in 33 eutherian genomes and evidence for conservation of numerous sequence elements in all 43 genomes as evolutionarily deep as zebrafish (Supplemental Fig. S3). The putative HOTAIR orthologs are encoded within the same genomic locus (between *HOXC11* and *HOXC12*) and have short conserved sequence elements. Several experimentally verified RNA structures in HOTAIR show signatures of evolutionary conservation (Somarowthu et al. 2015).

### Ortholog candidates are bona fide *roX* genes

Male-biased expression, RNA localization, and genetic rescue confirmed that the identified roX candidates were bona fide roX orthologs. We first assayed their expression in whole adult males and females by RT–PCR using species-specific primers for the roX ortholog candidates and a housekeeping mRNA (GPDH). In all 30 species tested, the roX1 and roX2 candidates displayed strong male-biased expression (Fig. 2C). These results indicate that our roX ortholog candidates, predicted from genomic sequence alone, are in fact male-biased RNA transcripts.

We next used RNA FISH (fluorescence in situ hybridization) to investigate the localization of roX1 and roX2 on *Drosophila busckii* polytene chromosome squashes. *D. busckii* was selected because of its basal position within the *Drosophila* subgenus, substantial evolutionary distance from *D. melanogaster* (diverged ∼40 million years ago) (Russo et al. 1995; Robe et al. 2010), and low homology with other roX orthologs. Notably, *D. busckii* roX2 paints the X chromosome in males but not females, and roX1 was not detected in either (Fig. 2D). This localization pattern matches that of *Drosophila mojavensis* and *Drosophila virilis* (like *D. busckii*, also in the *Drosophila* subgenus), in which roX2, but not roX1, coats the male X chromosome (Park et al. 2007).

Next we asked whether transgenic expression of *D. busckii* roX2 could rescue male lethality in *roX*-null *D. melanogaster*. As a positive control, transgenic expression of *D. melanogaster* roX2 rescued ∼75% of males (Fig. 2E). Notably, *D. busckii* roX2 rescued ∼20% of males, which, although modest, is substantially greater than the *roX*-null background (<0.01% male viability) (Deng et al. 2005). Complete structural disruption of the 3′ half of *D. melanogaster* roX2 abrogates male rescue (Ilik et al. 2013), but two chimeric fusions of *melanogaster–busckii* roX2 halves rescued males as robustly as the positive control (Fig. 2E). The enhanced rescue by the chimeric RNAs demonstrates the modular nature of structured repeats in lncRNAs (Quinn et al. 2014). Prior work showed that *roX*-null *D. melanogaster* males are best rescued by roX transgenes from *D. melanogaster*, followed by *Drosophila ananassae*, and then *D. willistoni*, suggesting that rescue efficiency decreases with increasing evolutionary distance (Park et al. 2008). The modest rescue by *D. busckii* roX2 fits this trend and confirms it as a bona fide roX2 ortholog. Because our bootstrapping strategy uses a chain of roX orthologs to iteratively bridge distantly related species, successful rescue by *D. busckii* roX2 implies that the intervening roX2 candidates are true orthologs as well.

### Conserved features of roX lncRNAs

Given that our search strategy begins by analyzing synteny, it is not surprising that most roX orthologs identified had conserved gene neighbors (Supplemental Fig. S2). In *D. melanogaster*, *roX1* and *roX2* loci are on the X chromosome, and, in all other species, the neighboring genes are also X-linked, suggesting that *roX* orthologs are similarly X-linked. This mirrors the finding that *Xist* orthologs in eutherians are always encoded on the X chromosome (Delgado et al. 2009). Using 5′-RACE and 3′-RACE, we showed that roX2 orthologs share a similar exon–intron gene structure, alternative splicing and polyadenylation pattern, and gene length (Supplemental Fig. S4). roX2 roXboxes are the most prominently conserved sequences in primary sequence, relative position, and orientation.

We found conserved structures in roX1 and roX2, including many novel structures as well as some with described functions (Supplemental Figs. S5, S6; Park et al. 2007; Park et al. 2008; Ilik et al. 2013; Maenner et al. 2013). For example, the roX1-D3 domain contains a stem–loop (IRB–RB) that was ultraconserved in every roX1 ortholog found (Supplemental Fig. S5C). Interestingly, another stem–loop in roX1-D3 is only present in the *Sophophora* subgenus and *Scaptodrosophila lebanonensis* but is absent in the *Drosophila* subgenus (Supplemental Fig. S5B) despite being a primary binding site for the DCC and important for roX1 function in *D. melanogaster* (Ilik et al. 2013; Quinn et al. 2014). Its presence in the outgroup species *S. lebanonensis* indicates that it was lost in the *Drosophila* subgenus (rather than gained in the *Sophophora* subgenus). Similarly, structures within roX1-D2 are lost in *D. willistoni* (Supplemental Fig. S5D). The absence of such important structures in these species may have consequences for roX1 function, which we explore below. We also found evidence for an ultraconserved structure (Supplemental Fig. S6B) in roX2 as well as complex structures in which two or more roXboxes compete for one intervening inverted roXbox, indicative of mutually exclusive alternative secondary structures (RB4–IRB and IRB–RB5) (Supplemental Fig. S6C). These structures are arranged on roX2 exon-3 in a similar configuration in all species (Supplemental Fig. S7).

### roX orthologs bind the X chromosome

To investigate the evolution of lncRNA–genome interactions, we mapped the genomic binding sites of roX1 and roX2 orthologs in four species: *D. melanogaster*, *D. willistoni*, *D. virilis*, and *D. busckii*. We chose these four species as representatives for the *Drosophila* genus’ diversity and distinct X chromosome karyotypes (Fig. 1B). The fruitfly genome consists of six chromosome arms, called Müller elements (MEs) A–F; the X chromosome in *D. melanogaster* is ME-A. However, the X chromosome in flies has undergone numerous karyotype reversals and ME fusions throughout evolution (Vicoso and Bachtrog 2015), such as the ME-A+D fusion in *D. willistoni* (Fig. 1B). Previous studies have found that newly evolved sex chromosomes can rapidly acquire DCC-binding sites through amplification of simple GA dinucleotide repeats that approximate the MSL recognition element (MRE) or domestication of MRE-bearing transposable elements (Alekseyenko et al. 2013; Ellison and Bachtrog 2013; Zhou et al. 2013).

**Figure 3.**
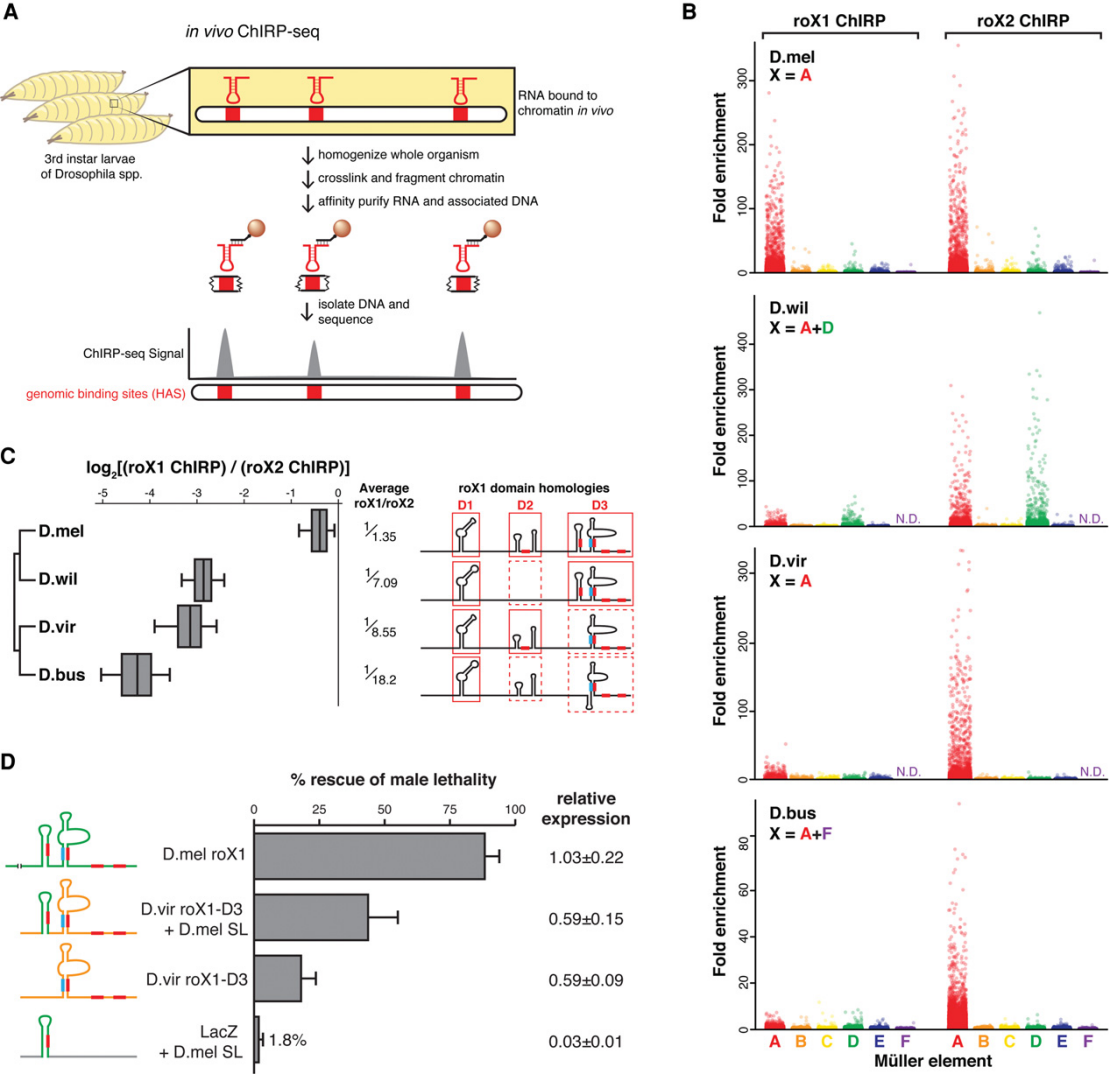
Genomic occupancy maps of roX orthologs highlight the loss of roX1–roX2 functional redundancy in other species. (*A*) ChIRP-seq identifies the genome-wide binding sites of an RNA target, performed directly from chromatin prepared from *Drosophila* larvae. (*B*) roX1 and roX2 signal enrichment (ChIRP/input) in 1-kb windows of MEs A–F in four *Drosophila* species. Signal is enriched on the X chromosome. roX1 enrichment is lower than roX2 in *D. willistoni* (D.wil), *D. virilis* (D.vir), and *D. busckii* (D.bus). (N.D.) No data, as no genome scaffolds aligned to ME-F. (*C*, *left*) The log ratio of roX1 to roX2 ChIRP signal at binding sites shows that roX2 is the dominant roX RNA in *D. willistoni*, *D. virilis*, and *D. busckii*. Average roX1/roX2 bias is shown as fraction. (*Right*) Known functional domains (red outlines), secondary structures, and roXboxes (filled red or blue rectangles) of roX1 are absent in *D. willistoni*, *D. virilis*, and *D. busckii*. Only *D. melanogaster* roX1 has a full complement of these repetitive elements. See also Supplemental Figure S5. (*D*) Rescue of male lethality in *roX*-null *D. melanogaster* males improves with the number of repetitive roXbox stem–loops. LacZ with *D. melanogaster* stem–loop (SL) rescues poorly, *D. virilis* roX1-D3 rescues modestly, and addition of the *D. melanogaster* stem–loop to *D. virilis* roX1-D3 further improves rescue, approaching the wild-type *D. melanogaster* roX1 rescue efficiency. Error bars and relative expression are as in Figure 2E.

We developed methods to perform in vivo ChIRP-seq (ChIRP and sequencing) directly from homogenized whole larvae. In ChIRP-seq, chromatin is cross-linked and fragmented, the target RNA and associated chromatin are affinity-purified with biotinylated antisense oligonucleotide probes, and the copurified DNA is sequenced (Fig. 3A). Thus, ChIRP-seq maps the in vivo genomic binding sites of a chromatin-associated RNA from endogenous interactions (Chu et al. 2011). Whereas ChIP-seq (chromatin immunoprecipitation [ChIP] combined with deep sequencing) in diverse species may require species-specific antibodies or transgenic epitope-tagging systems, ChIRP-seq in diverse species requires only new antisense oligonucleotide sequences that can be readily designed from lncRNA sequences regardless of how divergent they may be.

We performed roX1 and roX2 ChIRP-seq in the four species and mapped the reads to their respective genomes. We assigned scaffolds from each genome assembly to specific MEs based on coding sequence homology to *D. melanogaster* proteins, as done previously (Vicoso and Bachtrog 2015), and then calculated ChIRP signal enrichment (ChIRP/input) for each ME in 1-kb windows (Fig. 3B). We found that roX2 preferentially occupied the X chromosome in each species, including ME-D in *D. willistoni*. Interestingly, the tiny X-fused ME-F was not enriched in *D. busckii*, although this may be the result of the epigenetic silencing of ME-F and incomplete decay of the Y-fused ME-F (Vicoso and Bachtrog 2015; Zhou and Bachtrog 2015). The extensive roX2 binding on *D. willistoni* ME-D further supports the hypothesis that new X chromosomes evolve novel binding sites rather than modify or exchange DCC protein components (Alekseyenko et al. 2013).

Analysis of roX genomic occupancy indicated that roX1–roX2 functional redundancy has degenerated in some species. roX1 and roX2 ChIRP-seq is highly correlated for all species, indicating that, within each species, roX1 and roX2 bind the same loci, although with unequal potency (Supplemental Fig. S8). As expected, *D. melanogaster* roX1 and roX2 ChIRP-seq enriched for the X chromosome to approximately the same extent, but roX1 enrichment showed quantitative differences in the other species (Fig. 3B,C) despite equivalently effective capture of roX1 and roX2 RNAs in each species (data not shown). roX1 enrichment was 7.09-fold, 8.55-fold, and 18.2-fold weaker than roX2 in *D. willistoni*, *D. virilis*, and *D. busckii*, respectively (Fig. 3C). This is consistent with roX1's apparent absence on the X by RNA FISH in *D. virilis* and *D. busckii* (Fig. 2D; Park et al. 2007). The decreasing potency of roX1 in these species is correlated with the loss of stem–loops and roXboxes in domains D2 and D3 described above (Fig. 3C; Supplemental Fig. S5).

We tested the functional consequence of the loss of such repetitive structural elements in the roX1-D3 domain using transgenic rescue of *roX*-null *D. melanogaster* males (Fig. 3D). A transgene containing a single roXbox stem–loop from *D. melanogaster* roX1 embedded in bacterial LacZ mRNA rescued males poorly (1.8%). Although seemingly low, this level of rescue is ∼100-fold improved over *roX*-null flies (<0.01% male viability) (Deng et al. 2005), and thus such a stem–loop would confer a major selective advantage. Next, wild-type *D. virilis* roX1-D3 modestly rescues males (18%), consistent with its limited repertoire of roXbox stem–loops and modest X chromosome occupancy. Adding the *D. melanogaster* stem–loop to *D. virilis* roX1-D3 substantially improved male rescue (43%), approaching the rescue by the positive control, *D. melanogaster* roX1 (88%), which rescues to the same extent as roX1-D3 alone (Quinn et al. 2014). These findings suggest that, in the *Drosophila* subgenus (e.g., *D. virilis*), roX1 has vestigial function due to the loss of repeated structural elements; in flies like *D. melanogaster*, the observed roX1–roX2 functional redundancy results from the maintenance of such elements.

### roX-binding sites differ extensively across species

**Figure 4.**
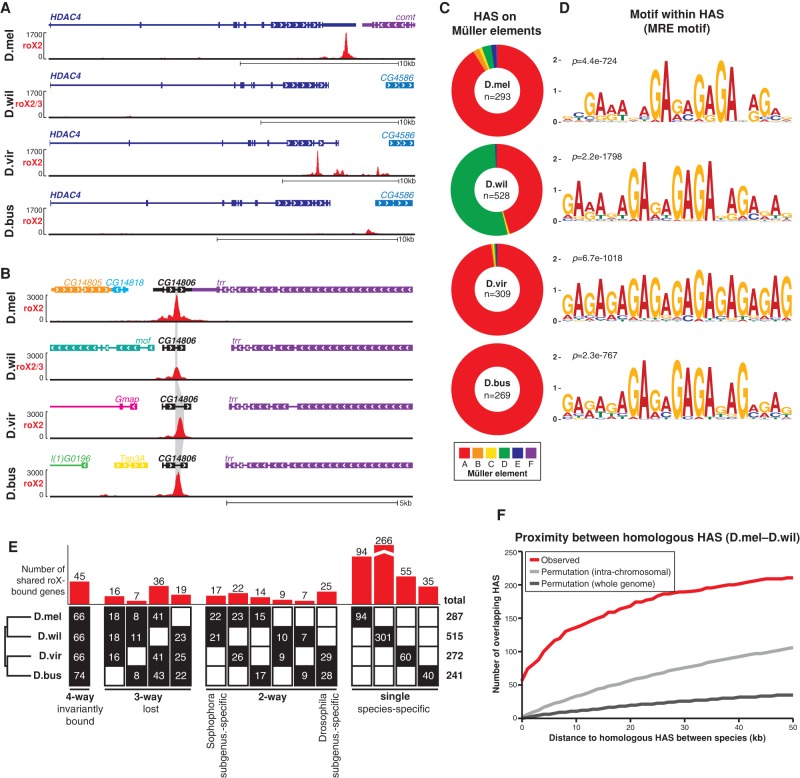
Evolutionary dynamics of roX-bound HASs. (*A*,*B*) roX2 ChIRP-seq tracks in representative windows on the X chromosome. (*A*) Some HASs are evolutionarily dynamic. The strong HAS in the 3′ untranslated region (UTR) of *D. melanogaster HDAC4* is absent or present elsewhere in other species. (*B*) Other HASs are evolutionarily conserved, such as the HAS in the last intron of *CG14806* (gray highlight). (*C*) roX2 ChIRP-seq identified hundreds of HASs on the X chromosome of each species. (*n*) Total number of HASs. (*D*) HASs contain GA dinucleotide repeats, characteristic of the MRE motif. (*E*) Gene-level conservation of HASs between four species. Forty-five genes are roX-bound (overlapping or neighboring a HAS) in all four species. *D. willistoni* has the most species-specific roX-bound genes because of its larger X chromosome. (*Top*) Number of shared roX-bound genes. (*Bottom*) Number of HASs within shared genes in each species. (*F*) Pairwise proximity of orthologous HASs between *D. melanogaster* and *D. willistoni*. About 60 HASs directly overlap in genomic lift-over (distance = 0); however, if an exact HAS homolog is lost (distance >0), another HAS is likely nearby. There are more overlapping HASs or nearby HASs (<30 kb) than expected by random permutation of HASs within each chromosome or over the whole genome. See also Supplemental Figure S12.

The high-resolution maps of roX RNA binding allowed us to trace the evolution and conservation of roX lncRNA–genome interaction at the level of chromosomes, genes, and individual DNA elements. roX-bound sites, known as HASs, are defined in *D. melanogaster* by joint binding of roX RNAs, MLE, and MSL2 (DCC proteins that directly bind roX) (Chu et al. 2011; Ilik et al. 2013; Straub et al. 2013). HASs contain a GA dinucleotide repeat sequence motif, called the MRE. The MRE motif is present on all chromosomes, yet the roX RNAs bind almost exclusively to a subset of MREs present on the X (Quinn et al. 2014). Close inspection of homologous genomic windows in the four species revealed that the positions of most roX-occupied HASs are evolutionarily dynamic (Fig. 4A), whereas a minority of HASs are at the same location in all species (Fig. 4B). HASs have conserved characteristics in each species. For example, there are hundreds of HASs on the X chromosome in each species, and *D. willistoni* has nearly twice as many HASs in accord with its approximately twofold larger X chromosome (Fig. 4C). The two HASs within the *roX1* and *roX2* loci were among the strongest binding sites and occupied by both roX RNAs in all species (data not shown), consistent with our previous report (Quinn et al. 2014). The few binding sites found on autosomes in *D. melanogaster* are reproducible (Quinn et al. 2014), and some are conserved in other species (Supplemental Fig. S9). In all species, the top enriched DNA sequence motif was a GA repeat matching the MRE motif in *D. melanogaster* (Fig. 4D), located at HAS centers (Supplemental Fig. S10). On *D. willistoni*’s ME-D, we did not find enrichment of any other sequences that would support alternative mechanisms of MRE accumulation (Supplemental Fig. S11); thus, the transposable element-taming mechanism reported in *Drosophila miranda* may be unique to *D. miranda* or species with more recently evolved neo-sex karyotypes (Ellison and Bachtrog 2013).

Detailed evolutionary analyses revealed that HASs are under selection for proximity but not precise location relative to genes. We counted the number of interspecies overlapping HASs at the level of genes or DNA elements. At the level of HAS-associated genes (defined as the nearest gene within 1 kb of a HAS), we found that a small proportion of genes is targeted in all four species (invariantly bound genes) (Fig. 4E). Instead, species-specific HAS-associated genes are the most abundant class (Fig. 4E, right), indicating poor conservation of the precise genes to which the DCC is targeted. Analysis of the distance between each HAS from one species and the nearest HASs in another species showed that HASs are significantly more likely to directly overlap or be present in the same chromosomal neighborhood than expected by chance alone (from randomly permuting HASs over their respective chromosomes or the whole genome) (Fig. 4F; Supplemental Fig. S12). The observed species-to-species distance between nearest homologous HASs is most enriched in local genomic neighborhoods up to ∼30 kb and then saturates. Thus, HASs exhibit a conservation pattern that is similar to transcriptional enhancers (Villar et al. 2015) but with a weaker level of conservation than some transcription factor-binding sites in closer related *Drosophila* species (He et al. 2011). This pattern suggests that if a specific HAS is lost in one species, another HAS likely arises nearby such that the number of and spacing between HASs do not change drastically.

### The host genome dictates the occupancy of transplanted roX RNAs

**Figure 5.**
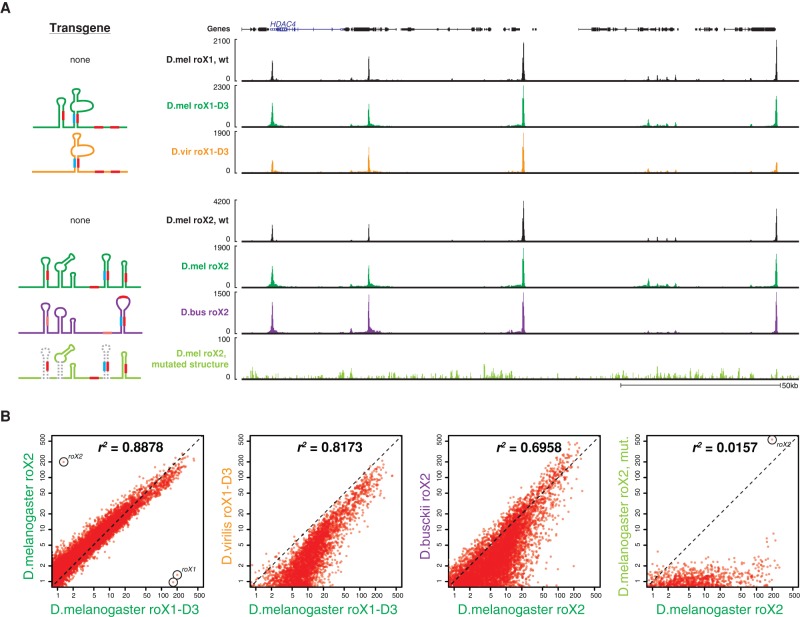
Transplanted roX RNAs from other species bind to *D. melanogaster*-specific binding sites. (*A*) ChIRP-seq of roX RNAs transplanted into *roX*-null *D. melanogaster*. (*Left*) Transgenic roX constructs showing structures and roXboxes. Dotted lines indicate mutated structures. (*Right*) A representative ∼150-kb window on the X chromosome showing ChIRP-seq tracks for each roX transgene relative to wild-type *D. melanogaster* roX1 and roX2 (black tracks) and transgenic *D. melanogaster* roX1 and roX2 (green tracks). Indicated *above* is the *HDAC4* locus, as in Figure 4A. *D. virilis* roX1 and *D. busckii* roX2 transgenes exhibit the same binding pattern as *D. melanogaster* roX RNAs, even at species-specific HASs. The mutated roX2 does not bind the X. (*B*) Pairwise comparisons of ChIRP-seq signal enrichment for each 1-kb window on the X chromosome. *roX1* and *roX2* loci are indicated as outliers due to genetic deletion of each locus. Correlation scores and overall enrichment are lower for *D. virilis* roX1 and *D. busckii* roX2 relative to *D. melanogaster* roX RNAs; log-scaled axes.

Differences in roX–genome interactions across species may arise from differences in the lncRNA, in the genome, or in both. To evaluate these possibilities, we performed ChIRP-seq on *D. virilis* roX1 and *D. busckii* roX2 expressed as transgenes in *roX*-null *D. melanogaster* (as in Figs. 2E, 3D). Both roX transgenes bound to the same sites as the *D. melanogaster* roX RNAs (Fig. 5A). For example, the *HDAC4* locus exhibits species-specific roX occupancy (detailed in Fig. 4A) but is always bound at the same (*D. melanogaster*-specific) site by transgenic *D. virilis* and *D. busckii* roX RNAs (Fig. 5A). As a negative control, we tested a *D. melanogaster* roX2 transgene with disrupted stem–loops, which fails to rescue *roX*-null males (Ilik et al. 2013); this mutant roX2 failed to bind HASs on the X. The similar binding patterns between these different species’ roX RNAs indicates that roX-binding sites are determined by the cognate sites in the host genome rather than by the roX RNA. However, both ChIRP-seq signal enrichment on the X and the correlation for *D. virilis* roX1 and *D. busckii* roX2 are lower than the *D. melanogaster* positive controls (Fig. 5B). Cross-species binding patterns are more concordant at strongly occupied regions but diverge more at medium and weakly bound sites, as indicated by the bimodal behavior of correlation. The weaker occupancy of the non-*melanogaster* roX transgenes provides one explanation for the partial rescue efficiency of these transgenes (Figs. 2E, 3D).

### Evolutionary origins of new roX-binding sites

**Figure 6.**
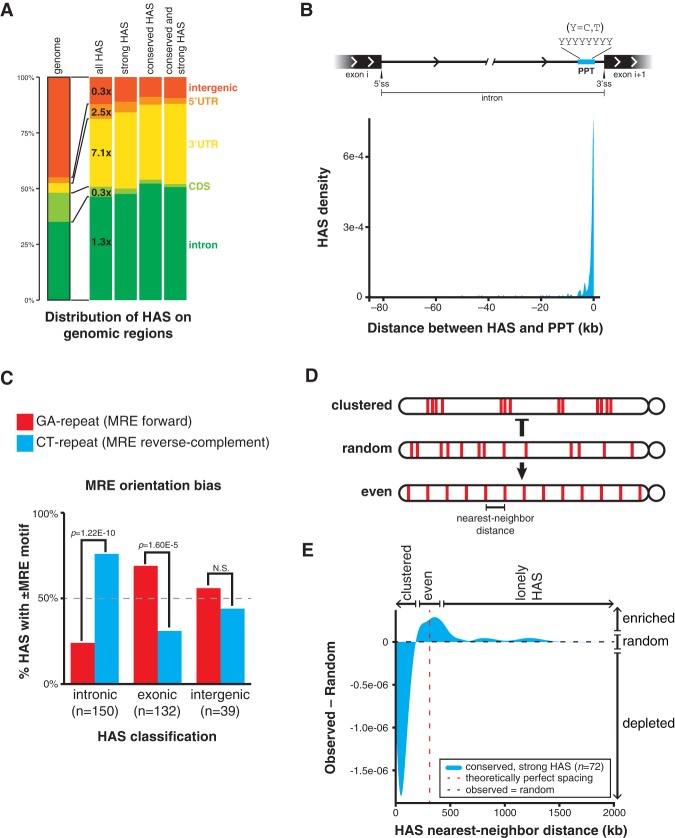
HASs exapt pre-existing regulatory signals and are selected for even spacing on the X chromosome. (*A*) HASs are enriched in genic, noncoding regions of the genome, primarily within introns and 3′ UTRs. HASs are subcategorized by strong roX occupancy and/or high evolutionary conservation. Fold enrichment over the genomic distribution is shown. (*B*) Intronic HASs are proximal to PPTs. See also Supplemental Figure S14A. (*C*) The MRE motifs within HAS classes exhibit significant and distinct orientation biases. Intronic HASs are biased in the reverse complement orientation (CT repeat), whereas exonic HASs are biased in the forward orientation (GA repeat); intergenic HASs have no bias. (*D*) Alternative HAS spacing models on the X chromosome. HASs may be clustered together, randomly spaced, or evenly spaced. The observed distribution is more even and less clustered than random. (*E*) The difference between the observed and random HAS (conserved, strong only) distributions on the X chromosome. The positive value near the theoretically perfect spacing distance indicates a more even spacing model relative to random spacing; conversely, the negative value at short distances indicates a less clustered model relative to random spacing. See also Supplemental Figure S13.

If the majority of HASs rapidly diverges throughout evolution, how do new HASs arise? We found that HASs are enriched in genic regions of the genome, especially within noncoding elements like introns and 3′ untranslated regions (UTRs) (Fig. 6A; Supplemental Fig. S13A). Enrichment on genic over intergenic regions is consistent with the idea that the DCC targets and regulates gene expression. Very few HASs are present in coding sequences, perhaps because the low-complexity MRE motif is not well tolerated in ORFs (Straub et al. 2013). As introns represent the most abundant location of roX binding (∼50%), we analyzed the position of HASs within introns. Notably, we found that HASs are proximal to the 3′ end of introns and are approximately threefold enriched at DNA encoding polypyrimidine tracts (PPTs), a C/T-rich splicing signal at the 3′ end of introns (Fig. 6B; Supplemental Fig. S14A). Approximately 20% of observed *D. melanogaster* HASs are within 100 base pairs (bp) of a PPT, versus ∼7% in a permuted background model (*P*-value = 1.77 × 10^−11^, K-S test). Conserved HASs are more enriched at PPT sites than species-specific HASs (28% vs. 15%). The association of HASs and PPTs also holds true for *D. willistoni* and *D. virilis* (Supplemental Fig. S13B,C). Moreover, HASs on *D. willistoni* ME-D are significantly enriched near PPTs (16% observed vs. 4% in permuted control; *P*-value < 2.2 × 10^−16^) (Supplemental Fig. S11) despite ME-D's ancestry as an autosome. Their homologous positions on the autosomal *D. melanogaster* ME-D are also enriched near PPTs (29% observed vs. 7% in permuted control; *P*-value = 3.90 × 10^−5^, K-S test). Thus, after the fusion of an autosome and the X, HASs can evolve from ancestrally autosomal PPTs (Supplemental Fig. S9). This suggests an alternative evolutionary pathway by which dosage compensation can colonize neo-X chromosomes (Ellison and Bachtrog 2013).

**Figure 7.**
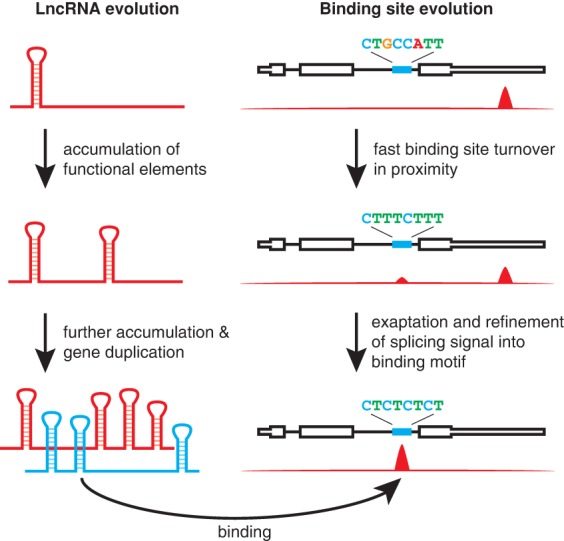
Models of roX and roX-binding site evolution. (*Left*) lncRNAs like roX can evolve function through the accumulation and maintenance of repetitive structures or sequence elements and gene duplication. (*Right*) lncRNA-binding sites are evolutionarily dynamic, losing function at one genetic element while gaining function nearby. The DCC and roX RNAs can coopt existing PPTs within gene introns, which are refined into the MRE sequence.

Importantly, the reverse complement of the GA repeat MRE motif is a CT repeat closely resembling the C/T-rich sequence of PPT, raising the hypothesis that PPT may serve as an abundant evolutionary source of MRE precursors. To test this hypothesis, we measured the strand bias in the MRE motif orientation relative to the direction of gene transcription. In the null hypothesis, MRE motifs in DNA would be independent of transcriptional direction and have no strand bias. Conversely, if MREs can arise from PPT, then the motif would be biased toward the pyrimidine-rich orientation. Indeed, intronic HASs are significantly overrepresented by the reverse complement MRE motif (CT dinucleotide repeat; *P*-value = 1.22 × 10^−10^, binomial test) (Fig. 6C; Supplemental Fig. S13D). HAS-containing introns are also more pyrimidine-rich (*P*-value = 4.21 × 10^−5^, K-S test) and substantially shorter (*P*-value < 2.2 × 10^−16^, K-S test) than typical introns (Supplemental Fig. S14B,C). Curiously, we also found that exonic HASs (primarily in 3′ UTRs) are significantly overrepresented by the forward MRE motif (GA dinucleotide repeat; *P-*value = 1.60 × 10^−5^, binomial test ) (Fig. 6C; Supplemental Fig. S13D). This bias further distinguishes PPT from other transcriptional units and reflects the slightly purine-rich environment of exons (Supplemental Fig. S14D,E). Taken together, these results suggest that some PPTs moonlight as MREs, having been coopted for dosage compensation and evolutionarily refined into true roX-binding sites (Fig. 7). This process of MRE refinement via PPT exaptation is clearly illustrated by the intronic HASs in genes *CG8097* and *Ns3*, for example (Supplemental Fig. S15).

Finally, we addressed potential selective pressures that drive the conservation of a subclass of HAS. We did not find any obvious genomic features or gene ontology terms for the genes near HASs with the highest evolutionary conservation and strongest binding signal. However, these “conserved, strong” HASs (72 in *D. melanogaster*) are more evenly spaced along the X chromosome than expected by chance alone (Fig. 6D,E; Supplemental Fig. S13E). The distribution of distances between nearest-neighbor HASs is different from permuted distributions with the same number of HASs and is enriched near the theoretically perfect spacing distance (the length of the X chromosome divided by the number of HASs). The “more even than random” placement of HASs thus maximizes HAS distribution along the X, which may therefore allow the DCC to spread as effectively as possible from a minimal number of HASs.

## Discussion

Using an integrative “nested homology” strategy based on phylogenetic conservation of synteny, microhomology, and RNA structure, we successfully identified 47 previously unknown roX lncRNA orthologs from 35 diverse flies. Despite very poor primary sequence homology, these distantly related roX orthologs have conserved structure and function and can suffice for dosage compensation in *D. melanogaster*. The discovery of these diverse roX orthologs permitted comparative analyses of RNA sequence, structure, and genomic interactions, revealing principles of lncRNA evolution and genomic targeting (Fig. 7). This integrative approach is likely applicable to trace the evolutionary dynamics of many lncRNAs that populate all kingdoms of life, as demonstrated by our description of the *HOTAIR* locus in species as diverse as humans and zebrafish (Supplemental Fig. S3). Indeed, we speculate that this strategy may have even greater success with *cis*-acting lncRNAs from vertebrate genomes; our strategy is parameterized on syntenic relationships, which are likely more conserved for lncRNAs that act in *cis* upon their gene neighbors (unlike roX and HOTAIR, which act in *trans*) (Rinn et al. 2007) in vertebrate genomes, which, relative to flies, have fewer chromosomal rearrangements that would otherwise break synteny (Ranz et al. 2001; Delgado et al. 2009). Furthermore, recent methods that reveal RNA structures in vivo (Spitale et al. 2015) should facilitate the systematic identification of lncRNAs by structural homologies, although, for the queries described here, computational analyses of RNA structure was sufficient. The search strategy described here differs from others in that it is targeted in scope, dynamically leverages new orthologs to improve search features, and requires only query genomes, whereas others require RNA-seq data, which are often sparse for nonmodel organisms (Necsulea et al. 2014; Hezroni et al. 2015).

Focal structures and repeated sequences emerged as key features for both the discovery and function of roX lncRNAs. In distantly related species, the roXbox stem–loops are often the only recognizable features linking roX RNA orthologs, and the number of the repeats correlates with the ability of roX1 orthologs to occupy the X chromosome. This insight allowed us to engineer designer lncRNA transgenes with one or more roXbox stem–loops, which functioned to varying degrees in vivo (Fig. 3C,D). This fits with the concept that lncRNAs evolve rapidly and can act as flexible scaffolds tethering together one or more functional elements (Guttman and Rinn 2012; Mercer and Mattick 2013). We found evidence for roX gene duplication in some species, producing lncRNA paralogs with support for divergence or partial loss of function of one paralog (akin to a lncRNA “pseudogene”) (Supplemental Fig. S1). Similarly, we showed that the complete roX1–roX2 functional redundancy observed in *D. melanogaster* is likely unique to certain species within the *Sophophora* subgenus, as roX1 orthologs in the *Drosophila* subgenus display limited localization to the X chromosome, which correlates with systematic loss of key structures and domains. The function, if any, of roX1 in the *Drosophila* subgenus may be addressed in the future by genetic disruption of one or both *roX* genes. Additionally, the discovery of roX1 and roX2 orthologs in more distantly related outgroup species may shed light on the evolutionary origin of these lncRNAs but would require more fully sequenced fly genomes. Did roX1 and roX2 originally evolve from an ancestral *roX* gene duplication event? Perhaps roX1–roX2 functional redundancy in certain flies allows divergent specialization in their regulatory programs or expression patterns, as with duplicated protein-coding genes that acquire divergent roles (Kellis et al. 2004). The repetition and refinement of functional elements may be a general principle in the evolution of some lncRNAs, as with roX and XIST (Fig. 7). Tracing the evolutionary patterns of key sequence or structural elements may shed light on the origin, diversification, and extinction of lncRNA genes.

We also describe the first comparative genomics analysis of the genomic binding sites of lncRNAs, which revealed the evolutionary constraints on lncRNA–genome interactions. By mapping the genome-wide occupancy of the roX RNAs, we found that roX-binding sites are always strongly enriched on the X chromosome, can turn over quickly, and are constrained in their chromosomal spacing pattern (Fig. 7). These features of evolutionary conservation are reminiscent of enhancer elements that bind transcription factors (He et al. 2011). The even distribution of binding sites on the X chromosome maximizes the coverage of the X while simultaneously minimizing the total number of HASs. This distribution pattern may enable the uniform and global regulation necessary for dosage-compensating the whole X chromosome. Moreover, prior studies in *D. melanogaster* suggested that roX can spread by spatial proximity in three dimensions rather than linearly (Grimaud and Becker 2009; Quinn et al. 2014), which is consistent with the even spacing pattern of binding sites. Our discovery of rapid turnover of individual roX-binding sites implies that new HASs are born frequently such that mutation of existing HASs does not compromise X chromosome dosage compensation. Furthermore, the evolutionary dynamism of HASs indicates that most individual binding sites are not essential for dosage compensation; in this way, DCC action is distributed rather than targeted, with the primary constraint dictating thorough coverage on the X. One abundant source of new HASs are intronic PPTs (Fig. 7; Supplemental Fig. S15), a feature of lncRNA targeting that was not previously appreciated, which would further facilitate the rapid invasion of the DCC to neo-X chromosomes. Thus, even when an autosome arm is fused to the X chromosome (forming a neo-X chromosome, as has happened repeatedly in evolution) (Vicoso and Bachtrog 2015), these newly X-linked genes may be readily targeted and subjected to dosage compensation. Exaptation of this splicing signal is an elegant strategy for dosage compensation because it parsimoniously encodes one function at the level of DNA (dosage compensation) and another at the level of RNA (splicing). Additionally, coupling dosage compensation machinery to DNA sequences encoding an RNA splicing signal may ensure that the dosage compensation machinery is targeted to bona fide genes that are transcribed and spliced.

Collectively, our data demonstrate the flexibility of lncRNA–genome interactions and suggest that they may drive epigenetic innovation in evolution. Comparative genomic studies of lncRNAs and their binding sites will be a powerful approach to address other questions about functions of the noncoding genome.

## Materials and methods

### LncRNA ortholog search strategy

The general principle for the lncRNA search strategy follows three primary steps: (1) initiation with a known lncRNA; (2) searching for closest-relative lncRNA orthologs using synteny, microhomology, and/or structure features; and (3) iteratively refining the search parameters with each newly discovered lncRNA ortholog. Steps 2 and 3 repeat by searching for the next closest related species’ lncRNA ortholog. In this way, one needs only knowledge of an initiating lncRNA from a single subject species (e.g., its sequence and neighboring genes) to query other sequenced genomes. In some instances, a sequenced genome is not necessary for discovering new lncRNA orthologs, as described below (i.e., analog search strategy based on degenerate PCR of syntenic protein-coding genes).

To initialize the search, we collected knowledge of roX1 and roX2 in *D. melanogaster* (and HOTAIR in *Homo sapiens*), specifically the neighboring syntenic genes, instances of repeated microhomology, and known secondary structures—both measured and predicted (Ilik et al. 2013; Quinn et al. 2014). For example, in *D. melanogaster*, *roX1* is flanked by protein-coding genes *yin* (upstream, sense) and *ec* (downstream, antisense); *roX2* is flanked by protein-coding genes *e(y)2* (upstream, antisense), *CG11695* (upstream, sense), and *nod* (downstream, sense) (Supplemental Fig. S2). Human HOTAIR is encoded in an ∼17-kb window between protein-coding genes *HOXC11* and *HOXC12* (Supplemental Fig. S3). To find repeated microhomologous sequence elements shared between roX1 and roX2, we searched for matching motifs shared between both RNAs using MEME (Bailey et al. 2009); this returned a collapsed roXbox sequence motif as a position-weight matrix. The structures of roX1 and roX2 have been measured or predicted previously (Ilik et al. 2013; Quinn et al. 2014), and we used NUPACK-predicted (Zadeh et al. 2011) structures for visual comparison with other lncRNA ortholog candidate structures. The structure module was not used to search for HOTAIR orthologs.

Using tBLASTn, we entered the amino acid sequences of syntenic protein-coding genes in *D. melanogaster* to search for orthologous protein-coding genes in the closest related species (i.e., within the melanogaster subgroup, such as *Drosophila simulans*). This then defined the genomic interval surrounding the candidate *roX* loci. Next, we matched the *D. melanogaster* roXbox motif to sequences within the synteny block using FIMO (Bailey et al. 2009). In each case, this elected a ∼500-bp window with a cluster of three to six high-scoring roXbox incidences corresponding to the roX1-D3 domain or roX2 exon-3 (Ilik et al. 2013; Quinn et al. 2014). We also computed the minimum energy structures within these windows using NUPACK (Zadeh et al. 2011) and visually compared the predictions to the structures in *D. melanogaster* roX1 and roX2, such as the repeated roXbox stem–loops (Ilik et al. 2013; Maenner et al. 2013; Quinn et al. 2014).

Using these new high-confidence roX1 and roX2 ortholog candidates from the expanded species list (i.e., all *melanogaster* subgroup flies), we updated the search parameters. Synteny remained unchanged, but we updated the microhomologous motifs with the additional roX1 or roX2 orthologs (thus improving the accuracy of the motifs and finding additional weakly conserved sites that could also be used for the orthology search). The structures for each of these species’ RNAs were collated for comparison in iterative search rounds. Equipped with these refined parameters, we expanded the search to more distantly related flies, such as those in the *melanogaster* group (e.g., *Drosophila takahashii*), thus iterating the search strategy and leveraging known phylogenetic relationships. For example, although *roX2* neighbors the *nod* gene in *D. melanogaster*, *roX2* neighbors *ari-1* in flies outside of the *melanogaster* subgroup (Supplemental Fig. S2); thus, we abandoned searching for syntenic regions around *nod* and instead focused on *ari-1*. With each new lncRNA ortholog candidate discovered, the search parameters became more and more refined, thus enabling the discovery and more distantly related orthologs.

In species lacking WGS assemblies, we used a PCR-based method to perform the synteny search. We designed degenerate PCR primers at conserved sequences in protein-coding genes expected to be syntenic with *roX* RNAs; if synteny was preserved, PCR yielded a DNA fragment, which we sequenced, and then we proceeded with the search strategy. By syntenic PCR, we found roX1 in *Drosophila nasuta* but not *Drosophila guttifera*, *Chymomyza pararufithorax*, or *Chymomyza amoena*; this suggests that either *ec*–*yin* synteny blocks have been disrupted or the syntenic protein-coding gene sequences are too divergent. We did not search for roX1 in *Drosophila paulistorum*, *Drosophila nebulosa*, *Drosophila saltans*, or *H. duncani*, as these flies were included for studying roX2–roX3 paralogy and lack WGS. roX2 could not be identified in *S. lebanonensis* because the *e(y)2–ari-1* loci are incomplete due to the low N50 of this genome assembly.

### Fly species and rearing

All fly stock species were sourced from the *Drosophila* Species Stock Center (http://stockcenter.ucsd.edu); the species stocks used here are listed in the Supplemental Material. All flies were raised on standard cornmeal–molasses medium or Wheeler-Clayton medium (*D. busckii* only) at room temperature unless specified otherwise.

For genetic experiments, the following stocks were obtained from the Bloomington Stock Center or were kindly donated: y^1^ w*; P{tubP-GAL4}LL7/TM3, Sb1 (Bloomington Stock Center, no. 5138), *w^1118^;P{da-GAL4.w^−^}3* (Bloomington Stock Center, no. 8641), and *roX1^SMC17A^, roX^2Δ^; CyO, hsp83-roX1* (Menon and Meller 2012).

### Genomic DNA and crude RNA extraction

Genomic DNA was extracted from whole adult mixed-sex flies using the Gentra Puregene kit (Qiagen); the gDNA was used for validation of roX loci sequences from WGS or synteny PCR with degenerate primers, as listed in the Supplemental Material. Crude RNA was extracted from whole newly eclosed male or female flies using TRIzol reagent (Life Technologies), treated with TURBO DNase (Life Technologies), and cleaned on RNeasy minicolumns (Qiagen); the crude RNA was used for RT–PCR expression analysis and RACE.

### Polytene squashes and RNA FISH

Polytene chromosome squashes were prepared from sexed wandering third instar larvae. Larvae were inverted, and the salivary glands were dissected. The glands were fixed in 3.7% formaldehyde + 1% Triton X-100 in PBS for 45 sec followed by 3.7% formaldehyde in 50% acetic acid for 2 min. The glands were transferred to 15 µL of 50% acetic acid and 17% lactic acid on a siliconized coverslip. Polytene chromosomes were squashed beneath a polylysine slide, flash-frozen in liquid nitrogen, uncovered, and dehydrated in 100% ethanol for 30 min. Finally, slides were washed twice in PBS before proceeding to single-molecule FISH staining and imaging on a fluorescent microscope, according to the Stellaris protocol (Biosearch Technologies). Single-molecule FISH probes are listed in the Supplemental Material.

### RT–PCR, RACE, and synteny PCR

Oligo(dT)-primed cDNA libraries were made from crude RNA extract from each species using a SuperScript III first strand synthesis system (Life Technologies). RT–PCR was performed using species-specific primers against roX1, roX2 (and roX3, when applicable), and GPDH and amplified for 30 cycles. 5′-RACE and 3′-RACE were performed using the GeneRacer kit (Life Technologies) starting from crude RNA. Syntenic PCR was performed from genomic DNA using degenerate primers designed against conserved syntenic genes or regions. See the Supplemental Material for the lists of all primers used.

### Sequence identity and structure modeling

Sequence conservation was calculated using Clustal Omega 1.2.1 (DNA MSA, standard settings) for each roX1 and roX2 (and roX3, when applicable) relative to two scrambled sequences independently generated by scrambling the *D. melanogaster* sequence. The percentage sequence identity was calculated for every pairwise comparison. The lower limit of sequence homology is the average percentage sequence identity between roX and scrambled sequences (36%, not the theoretical 25%, due to nucleotide overrepresentation). Pairwise percentage sequence identity was plotted between 36% and 100%. NUPACK (Zadeh et al. 2011) was used to predict local RNA secondary structures in roX1 and roX2.

### Genetic experiments

Fly work was done essentially as described (Ilik et al. 2013; Quinn et al. 2014). Briefly, all *roX1* and *roX2* constructs were cloned into pUASattB vector, and transgenic flies were generated using φC31 integrase-mediated germline transformation as described (Groth et al. 2004), injecting y^1^ M{vas-int.Dm}ZH-2A w*; PBac{y^+^attP-3B}VK00033 embryos. To score male-specific lethality rescue, *roX1^SMC17^A, roX^2Δ^;; daGAL4* or *roX1^SMC17A^, roX^2Δ^;; tubGal4/TM6Tb* virgin females were crossed to UAS-roX1* and UAS-roX2* males, respectively, and allowed to develop at 25°C. roX1* denotes the transgenic construct; namely, *D.mel roX1* (full-length), *D.vir roX1-D3*, and *D.vir roX1-D3* + *D.mel SL* in Figure 3D. roX2* denotes the transgenic construct; namely, *D.mel roX2-exon3*, *D.bus roX2-exon3*, *D.bus roX2-5*′ + *D.mel roX2-3*′ (chimera 1), and *D.mel roX2-5*′ + *D.bus roX2-3*′ (chimera 2) in Figure 2E.

Male and female adult flies from at least three independent crosses were counted daily for a period of 10 d from the start of eclosion without blinding. The total number of non-Tb males was divided by the total number of non-Tb females that eclosed during the 10-d period, which was used as an internal control for 100% viability.

Gene expression analysis was done as described (Ilik et al. 2013; Quinn et al. 2014). Briefly, three to four third instar larvae were homogenized in Trizol, and total RNA was extracted using the Direct-zol kit (Zymo). RNA was reverse-transcribed with SuperScript III and random hexamers (Life Technologies). Relative expression values were calculated using the 2^ΔΔCt^ method, using PFK mRNA as an internal control.

### In vivo ChIRP-seq

ChIRP-seq protocol was adapted from Chu et al. (2011), and chromatin preparation from larvae was adapted from Soruco et al. (2013) and Alekseyenko et al. (2006). First, 1.0 g of mixed-sex wandering third instar larvae (between ∼300 and 1500 larvae, depending on size) was collected, washed in PBS, flash-frozen in liquid nitrogen, and pulverized into a fine powder using a mortar and pestle under liquid nitrogen. (For mutant ChIRP-seq experiments, 150 mg of male larvae was collected.) Next, the powder was reconstituted in 40 mL of cold PBS with protease inhibitor cocktail (Roche) and homogenized in a dounce tissue grinder (Kimble Chase). The homogenized material was passed through a 100-µm nylon SteriFlip filter (Millipore) and immediately fixed in 1% formaldehyde by nutation for 20 min at room temperature. Fixation was quenched with 5% volume of 2.5 M glycine for 5 min. The fixed material was pelleted by centrifugation at 3800 rpm for 30 min at 4°C and washed with cold PBS. The pellet was resuspended in 2 mL of cold swelling buffer (0.1 M Tris at pH 7.0, 10 mM KOAc, 15 mM MgOAc) supplemented with 1% NP-40, protease inhibitor, and Superase-In (Ambion); incubated for 10 min on ice; and dounced for 2 sec with a handheld motorized homogenizer (Argos) fitted with 1.5-mL tube pestles (VWR). Material was pelleted by centrifugation at 5000 rcf for 10 min at 4°C and washed in cold PBS. Next, the material was further fixed with 3% formaldehyde in PBS by nutation for 30 min at room temperature; cross-linked material was pelleted by centrifugation at 3500 rpm for 30 min at 4°C, washed in PBS, and pelleted. Cross-linked material was resuspended in 7 mL of nuclear lysis buffer supplemented with protease inhibitor cocktail and Superase-In and then solubilized and sheared by sonication using a Covaris E-series focused ultrasonicator (850 µL per tube, 4°C water bath, 5% duty cycle, 140 PIP, 60 min total). Nucleic acid shearing was confirmed by agarose gel electrophoresis. The resulting chromatin was clarified by spinning at maximum speed on a tabletop minifuge for 10 min at 4°C; the soluble chromatin fraction was collected and flash-frozen in liquid nitrogen or immediately used for ChIRP.

ChIRP was performed as described in Chu et al. (2011). ChIRP oligos were designed against roX1 and roX2 RNAs from each species using the Stellaris single-molecule FISH oligo designer (Biosearch Technologies). ChIRP oligos and pools are listed in the Supplemental Material. The DNA fraction from each ChIRP experiment and inputs were purified, and libraries were constructed using the NEBNext DNA library preparation kit (New England Biolabs). Sequencing libraries were barcoded using TruSeq adapters and sequenced on HiSeq or NextSeq instruments (Illumina) using single-end 50-bp reads. Reads were processed using the ChIRP-seq pipeline (Chu et al. 2011).

### Peak calling, filtering, and motif analyses

Peaks were called from the merged even–odd roX2 ChIRP-seq tracks using MACS2 (no peak model, 150-bp extension size, summit calling enabled). Called peaks were filtered by their significance [−log_10_ (*q*-score) ≥3000; ≥8000 for *D. willistoni*] and enrichment (ChIRP/input ≥20). Sequence motifs were discovered using MEME in 500-bp windows centered on peak summits (ZOOPS; 21-bp window). The central location of each motif occurrence was determined using CentriMo (Bailey et al. 2009).

### Signal enrichment analysis

ChIRP-seq signal enrichment was calculated for every 1-kb window of the genome as the sum of signal from roX1 or roX2 ChIRP divided by the input signal from the same window. The enrichment was then plotted as grouped by ME assignments (see below). The 5-kb windows around the *roX1* and *roX2* loci were excluded due to the possibility of direct genomic DNA recovery by antisense ChIRP oligos. To calculate the roX1 versus roX2 signal bias, the ChIRP-seq signal ratio was calculated for each peak. Box and whisker plots represent the 95/75/50/25/5 percentiles, plotted on a log_2_ scale, and the fractional bias represents the median roX1 to roX2 bias.

### Genome assemblies

All genome builds were obtained from FlyBase (http://www.flybase.org) with the following exceptions: *Drosophila americana* (genome assembly downloaded from the Jorge Vieira laboratory Web site, http://evolution.ibmc.up.pt), *Drosophila suzukii* (Spotted Wing FlyBase; Chiu et al. 2013), and *Drosophila mauritiana* (Nolte et al. 2013). For *D. busckii*, we downloaded the raw WGS reads from the NCBI Short Read Archive (SRP021047). We assembled the genome as described (Vicoso and Bachtrog 2015) with the exception of using SOAPdenovo2 (Luo et al. 2012). Only scaffolds >1 kb were retained. Our *D. busckii* assembly is available on Gene Expression Omnibus (GEO; accession below) with assembly statistics in the Supplemental Material.

### Protein-coding gene annotation

We obtained all genome annotations from FlyBase, except for *D. busckii*. The genome annotation information is available from GEO (accession below). For *D. busckii*, we annotated putative protein-coding genes using homology transfer of *D. melanogaster* protein-coding sequences. The homology transfer was based on the genBlastA pipeline (She et al. 2009), which uses BLAST to find high-scoring pairs (HSPs) between *D. melanogaster* and *D. busckii*. The parameters used in running genBlastA were -p T -e 1e-1 -g T -f F -a 0.6 -c 0.4 -d 100000 -r 10 -s 0.

For each ChIRP-seq peak, we used intersectBed (BEDTools suite) (Quinlan and Hall 2010) to find the genomic features to which the peak summit belongs based on FlyBase annotations. A small fraction of genomic features overlap, and, as such, some peak summits were double-counted (e.g., a summit could be in the intron of one transcript and the exon of another).

### ME annotation

We implemented a pairwise genome alignment pipeline based on LASTZ (Chiaromonte et al. 2002) and the University of California at Santa Cruz (UCSC) tool set, following a protocol from UCSC (http://genomewiki.cse.ucsc.edu). We compared our alignment of *D. melanogaster* and *D. virilis* with the liftOver file downloaded from UCSC and confirmed that they are virtually identical.

Based on the pairwise genome alignment, we calculated an empirical similarity score for each scaffold of *D. virilis*, *D. willistoni*, and *D. busckii* and each ME of *D. melanogaster*. The score is defined as the chain score between the scaffold in the first species and the ME in the latter divided by the total chain score of the scaffold and all MEs. We applied a stringent cutoff of 0.85 to reliably assign a scaffold to a ME. This assigns most scaffolds that are >1 kb. For very long scaffolds below this cutoff, we manually inspected the empirical score and homology information of protein-coding genes on the scaffolds and the correspondent ME. For example, we assigned *D. willistoni* scaffold scf2_1100000004963 to ME-A (similarity score 0.797, protein homology percentage 90%; i.e., 90% proteins homologous to *D. melanogaster* ME-A).

### Gene-level and element-level peak overlaps

For each ChIRP-seq peak, we assigned a gene association if the peak summit was within 1 kb. For *D. virilis*, *D. willistoni*, and *D. busckii*, since the UTRs were not annotated, we included a typical length of 200 bp or 500 bp for the 5′ UTR and 3′ UTR, respectively. After this assignment, starting from each peak in each species, we asked whether it had related peaks in other species based on the orthology information annotated in FlyBase for *D. melanogaster* genes and *D. virilis* or *D. willistoni* genes and annotations for *D. busckii* (described above). For a peak in species *A*, if its associated genes contained a gene ortholog associated with a peak in species *B*, the peak was regarded as gene-wise conserved between species *A* and *B*. Otherwise, the peak was regarded as species-specific.

We also investigated the conservation of the genomic positions of a ChIRP-seq peak in different species based on our pairwise whole-genome alignment. Specifically, for each peak in a species *A*, we used the liftOver tool to find its homologous position in species *B*. If the position overlapped with a peak in species *B*, it was regarded as conserved. We studied the peak turnover by allowing the homologous position to be within variable distance of a peak in species *B*. We observed that if the homologous position did not overlap with a peak in species *B*, then there was often a peak present nearby. We compared this distribution to random chance by permuting the peaks on species *B* within the same chromosome or across the whole genome using shuffleBed.

### Peak to PPT summit calculation

For each intron, we obtained its sequence and predicted the positions of PPTs within the intron by using the online tool SVM-BPfinder (Corvelo et al. 2010). We then implemented an algorithm to select the most likely PPT for each intron by adding a penalty score that increased with the distance to the 3′ splicing site. Specifically, if the distance was <40 bp of the 3′ splicing site, the penalty score equaled 0 but increased by 0.02 per base. For all ChIRP-seq peaks, we calculated the directional distance to its nearest PPT, upstream or downstream. We then permuted the position of each ChIRP-seq peak within the same chromosome and calculated again the directional distance of a random peak to its nearest PPT. We compared the two distributions by using a two-tailed K-S test. We also counted the percentage of observed or random peaks within 100 bp of a PPT.

### MRE motif orientation bias analysis

We used MEME to identify the position and orientation of the best MRE motif within each ChIRP-seq peak of each species. The positions of the MRE motif were used to annotate which genomic feature the peaks were then assigned (e.g., coding sequence, intron, etc.). The motif orientation instances (+/−) were counted for each category of genomic features, and a binomial test was used to quantify the differences.

### Chromosome spacing analysis

We calculated the distance for each peak summit to its nearest neighbor. If ChIRP-seq peaks were perfectly evenly distributed on a chromosome, the nearest-neighbor distance would be the length of the chromosome divided by the total number of peaks; if all peaks were clustered, the nearest-neighbor distances would approach 0. We also simulated the random distance distributions by shuffling the peaks to random positions within the chromosome.

We defined a subset of strong peaks (enrichment >50 and log_10_ (*q*-value) >10,000; >20,000 for *D. virilis*) or conserved peaks (shared in at least one other species). We further defined a subset of strong and conserved peaks as the intersection of these two sets. We calculated the above analysis of nearest-neighbor distance using this subset of peaks. The difference between observed and the random distributions of nearest-neighbor peak distances is plotted.

### Accession codes

The raw sequencing reads from each ChIRP-seq experiment (*.fastq), the mapped and merged ChIRP-seq and input tracks (*.bedGraph and .bigWig), called ChIRP-seq peaks (*.bed), ME assignments (*.xlsx), *D. busckii* genome assembly and annotations, and roX1 and roX2 sequences can be accessed at GEO (accession no. GSE69208). All raw and processed sequencing data can be accessed in NCBI's GEO through accession number GSE69208.

## Supplementary Material

Supplemental Material
