# Supplementary material for: Rapid evolutionary turnover underlies conserved lncRNA–genome interactions
Source: Genes Dev. 2016 Jan 15;30(2):191–207. doi: 10.1101/gad.272187.115 (PMC4719309; doi:10.1101/gad.272187.115)
Supplement: Supplemental Material [file supp_30_2_191__index.html]

Supplemental Material 

# Rapid evolutionary turnover underlies conserved lncRNA–genome interactions

## Supplemental Material

**Files in this Data Supplement:**

- Supp Figures.pdf
- Supp Material.xlsx
