# Supplementary material for: Rapid evolutionary turnover underlies conserved lncRNA–genome interactions
Source: Genes Dev. 2016 Jan 15;30(2):191–207. doi: 10.1101/gad.272187.115 (PMC4719309; doi:10.1101/gad.272187.115)
Supplement: Supplemental Material [file supp_30.2.191_SuppFigures.pdf]

Supplemental Figure Legends

Supplemental Figure S1. *D. willistoni* has three roX RNAs resulting from a *roX2* gene duplication event in the *willistoni-saltans* clade.

(A) The *roX2–roX3* locus of *D. willistoni* and its relatives. Our ortholog search in *D. willistoni* identified a third roX homolog candidate adjacent to *roX2*. Using synteny PCR from *e(y)2* and *ari-1* or *roX2*, we cloned and sequenced the *roX2–roX3* locus in four relatives of *D. willistoni*. The roX2–roX3 pairs are present in *D. willistoni, D. paulistorum, D. nebulosa*, and *D. saltans*, but absent in the sister species *Hirtodrosophila duncani*, indicating that roX3 arose after the divergence of *H. duncani* and the concestor of the *willistoni-saltans* clade. The *D. melanogaster roX2* locus is shown for reference.

(B) Sequence identity between roX2 and roX3 in five species. The roX2 and roX3 orthologs share relatively high sequence identity both between species and between roX2–roX3 pairs, indicating that roX2 and roX3 are likely paralogs that resulted from a whole gene duplication event.

(C) roX2 and roX3 RNA expression. roX2 and roX3 orthologs are male-biased transcripts in the *willistoni-saltans* clade, as is roX2 in *H. duncani*. Expression bias between roX2 and roX3 is species-specific; for example, roX2 expression is nearly undetectable in *D. paulistorum*, yet roX2 expression is higher than roX3 in *D. nebulosa*. GPDH is used as a sex-independent control.

(D) The roXboxes (RB1, RB4-6; red) and inverted roXbox (IRB; cyan) of roX2–roX3 orthologs are conserved horizontally between species and vertically between roX2–roX3 paralogs.

(E) The stem-loop structure at the 5'-end of roX2 and roX3 orthologs is conserved between species and between roX2–roX3 paralogs. This structure contains RB1 (red circles). *H. duncani* roX2 has an extended stem-loop.

(F) ChIRP-seq was used to map the chromatin occupancy of the roX RNAs in *D. willistoni*. The *roX2–roX3* locus is shown with signal from roX1 and roX2/3 ChIRP-seq and input DNA. The *roX2* and *roX3* loci exhibit a very similar pattern of roX binding, indicating that the HAS at these paralogous loci are also conserved.

Supplemental Figure S2. roX1 and roX2 ortholog synteny is maintained across evolution.

(A) In *D. melanogaster*, *roX1* is flanked by protein-coding genes *yin* (upstream, sense) and *ec* (downstream, antisense). In nearly all other species, these flanking genes are maintained in position and orientation; one such exception is *D. ananassae*, in which an intrachromosomal rearrangement replaced the downstream neighboring gene *ec* with *CG5254*. For brevity, only representative species from major fly clades are shown. Scale bars, 5kb.

(B) Similarly, in *D. melanogaster*, *roX2* is flanked by protein-coding genes *CG11695* (upstream, sense), *e(y)2* (upstream, antisense), and *nod* (downstream, sense). Synteny is not always perfectly preserved, however; though *nod* is downstream of *roX2* in the *melanogaster* subgroup, *ari-1* is downstream in all other flies outside the *melanogaster* subgroup. Thus, the *roX2–ari-1* synteny block is ancestral, and an intrachromosomal shuffling event in the ancestor of the *melanogaster* subgroup replaced *ari-1* with *nod* at the *roX2* locus. For this reason, when searching for roX2 orthologs in species outside the *melanogaster* subgroup, we instead used *ari-1* as a flanking gene instead of *nod*. Using *nod* in any search outside of the *melanogaster* subgroup would prove unproductive, since this syntenic relationship is not maintained. This instance highlights the importance of using phylogenetic relationships as a guide in the search strategy. When performing the synteny search in species lacking WGS, such as *D. nasuta*, we anticipated that *D. nasuta* might have similar gene order to its closest relative with a sequenced genome, *D. albomicans*. We designed degenerate primers at evolutionarily conserved sites in the four flanking protein-coding genes (*ec* and *yin*, and *e(y)2* and *ari-1*, for the *roX1* and *roX2* loci, respectively). In both cases, PCR yielded a fragment of roughly the expected intervening size, which we then sequenced.

Supplemental Figure S3. The lncRNA ortholog search strategy found the *HOTAIR* locus in 43 diverse vertebrate species.

(A) HOTAIR is a lncRNA that was discovered in human and has been described in mouse. It is transcribed from the *HOXC* locus, flanked by and antisense to the protein-

coding genes *HOXC11* and *HOXC12*, which are highly conserved across vertebrate genomes.

(B) We searched for the *HOTAIR* locus in 43 diverse vertebrate species from primates down to zebrafish (which diverged ~400Mya). We used the synteny module of the lncRNA ortholog search strategy, initiating with knowledge of only human HOTAIR. We found *HOXC11* and *HOXC12* on the same genomic scaffold in a window of ~21kb, suggesting that the syntenic relationship with *HOTAIR* is maintained. In at least six species for which there are expressed sequencing tags (ESTs), there was an EST in the intergenic space between *HOXC11* and *HOXC12*, mapping to the location where *HOTAIR* would be expected. This suggests that an intergenic lncRNA – presumably the HOTAIR ortholog – is encoded at this locus. K, known HOTAIR lncRNA; Y (cyan), HOTAIR lncRNA ortholog candidate identified.

(C) Using the motif discovery algorithm MEME, we searched for instances of microhomology in the putative HOTAIR loci. At least six instances of focal microhomology were found, represented as colored boxes and corresponding to specific positions mapped to human HOTAIR. All six elements (red through purple) are conserved across all eutherian mammals, while one (yellow) has much deeper evolutionary conservation and can be found in zebrafish. Despite monotremes' closer relation to eutherian and metatherian mammals, many of the sequence elements found in HOTAIR are absent. Pale boxes with dotted outline indicate species for which not all elements within a microhomologous sequence motif are present (i.e. incomplete microhomology).

(D) Sequence motifs for the conserved HOTAIR elements. One motif (red) is found at the promoter for the human HOTAIR lncRNA, and its conservation suggests that this promoter is conserved in eutherian and metatherian mammals. Similarly, a splice site (cyan) is conserved in eutherian and metatherian mammals. Together, the conservation of these transcription- and splicing-associated signals suggests that this locus in other species is also transcribed and spliced. Whether these are microhomologous elements are functional at the DNA level (e.g. transcription factor binding sites, enhancers, etc.) or at the RNA level (RNA-binding protein sites, RNA processing sites, microRNA targets, etc.) and their importance to HOTAIR function remains to be validated.

Supplemental Figure S4. roX2 orthologs have similar gene model, roXbox content, and roXbox sequences.

(A) We performed 5'- and 3'-RACE (rapid amplification of cDNA ends) on a selection of 16 diverse roX2 orthologs to define their gene structure, including transcriptional start sites (TSS; green flags), alternative splicing (gray boxes and dashed lines), and polyadenylation sites (PAS; red arrowheads). These 16 species were selected for their diversity and representation of major fly clades. *D. melanogaster* roX2 is known to have numerous alternative splice forms (Park et al. 2005). The major isoform of *D. melanogaster* roX2 consists of a short first exon (~30bp), an intron (~600bp, called "exon 2" despite being an intron), and exon 3 (~500bp, contains roXboxes and described secondary structures); however, many minor isoforms are generated by alternative splicing within "exon 2".

(B) RACE showed that all orthologs analyzed share a similar gene structure: a major isoform consisting of exon 1–exon 3 with minor isoforms from alternative splicing of "exon 2". TSS within the short first exon vary, as do PAS within exon 3, occurring most commonly 3' of roXbox-4, -5, or -6. The relative positions, number, and orientation of roXboxes (RB, red blocks) and inverted roXboxes (IRB, cyan blocks) are consistent across most species. Here, graphical alignment is relative to RB5, the most highly conserved of these sequences.

(C) Indeed, the RBs and IRB are the most highly conserved sequence elements in roX2 orthologs. The 8-nucleotide RB or IRB motif is underlined. Motifs were calculated over all discovered roX2 (and roX3) orthologs. Other weakly conserved sequences occur within "exon 2" and are likely implicated in alternative splicing (not shown).

Supplemental Figure S5. roX1 orthologs have similar secondary structures, but the Drosophila subgenus lacks a critical stem-loop in domain D3 and a structure in D2 is lost in *D. willistoni*.

(A) The secondary structures of roX1 in *D. melanogaster* are organized around three primary functional domains: D1, D2, and D3 (red boxes). D3 contains four RBs and one IRB (red and cyan blocks, respectively), and D2 contains one RB. The indicated

structures and their conservation are shown in panels **B-E**. All structures are drawn 5'-to-3', left-to-right. For brevity, only representative species from major clades are shown.

(B) The stem-loop within D3 of roX1 contains a RB1 (red highlight) and is conserved in the Sophophora subgenus, but both the roXbox and the structure are absent in the Drosophila subgenus. The stem-loop and RB1 are present in the outgroup species, *S. lebanonensis*. See also **Fig. 3C**.

(C) The IRB-RB2 (red highlight) stem-loop within D3 of roX1 is conserved across all *Drosophila* species.

(D) The stem-loops within domain D2 are absent in *D. willistoni*, which has no apparent D2 domain. Most species in the Drosophila subgenus also lack the first stem-loop structure in D2 (not shown).

(E) The primary stem of domain D1 is found in all species, and is proximal to the MRE within the *roX1* locus (which acts as an active HAS in all species investigated).

Supplemental Figure S6. roX2 orthologs have similar secondary structures, including a conserved pair of alternative secondary structures between RB4, IRB, and RB5.

(A) The structure of model *D. melanogaster* roX2, all within exon 3. The indicated structures and their conservation are shown in panels **B-C**. All structures are drawn 5'-to-3', left-to-right. For brevity, only representative species from major clades are shown.

(B) The stem-loop at the 5'-end of roX2 contains a roXbox (RB1) and is conserved across all Drosophila species.

(C-D) Two alternative structures are ultraconserved across every species, and involve RB4, IRB, and RB5. RB4 and RB5 compete for the single intervening IRB to form two alternative and mutually exclusive structures, the RB4–IRB conformation or the IRB–RB5 conformation. Both structural forms are shown simultaneously here, competing for the central IRB element, in cyan (note: structure cartoon does *not* depict an RNA triplex).

Supplemental Figure S7. The structural organization of roX2 exon 3 is conserved. RB (red blocks) and IRB (cyan blocks) fold into stem-loops (colored arcs; circle plot). The P1 stem-loop (red arcs) and RB4–IRB–RB5 alternative structures (cyan, blue, and purple) are the most conserved structures; see also **Supplemental Fig. S6**. For brevity, only representative species from major clades are shown.

Supplemental Figure S8. Within each species, roX1 and roX2 have the same binding sites, though with different absolute affinities. In all species, roX1 and roX2 bind to the same loci, though some are biased towards roX2. The signal from roX1 or roX2 ChIRP-seq in 1kb windows of the X-chromosome (ME-A in all species, plus ME-D in *D. willistoni*) was integrated, and plotted against one another. There is high correlation between roX1 and roX2 signal, especially for *D. melanogaster, D. willistoni*, and *D. virilis* with greatly diminished correlation in *D. busckii*. The roX1 signal is substantially lower than roX2 signal for all species except *D. melanogaster*, indicating a bias towards roX2 as the dominant roX homolog in these species (see **Fig. 3C**; note that *x*- and *y*-axes are not equally scaled). The 5kb windows surrounding the *roX1* and *roX2* loci were excluded due to direct ChIRP oligo–genomic DNA hybridization and recovery.

Supplemental Figure S9. roX RNAs bind to dozens of autosomal sites, some of which are conserved X-linked HAS in *D. willistoni*.

(A-B) Two such HAS at the TSS / promoters of autosomal genes (on ME-D) are shown, *RasGAP1* and *Sox21b*. These genes are autosomal in *D. melanogaster* (on ME-D), but X-linked in *D. willistoni* (due to its ME-A+D fusion). The HAS at the TSS / promoters of these two genes are conserved between *D. melanogaster* and *D. willistoni*; however, in *D. willistoni* an additional HAS is immediately downstream from the *RasGAP1* stop codon (presumably in the 3'UTR). This suggests that preexisting autosomal binding sites may also serve as HAS after neo-sex chromosome karyotype fusions.

(C) The autosomal HAS in *D. melanogaster* are present on all autosomes (ME-B through -F), and are predominantly at TSS / promoters (as opposed to the intronic and 3'-UTR bias of X-linked HAS). These autosomal HAS are reproducible across roX1 and roX2

ChIRP-seq and between different ChIRP-seq experiments in different cell types (Quinn et al. 2014). Interestingly, some of these genes have male-specific or male-biased expression, such as *chinmo*, *Sox21b*, and *dac* (not shown), suggesting that male-biased autosomal genes may coopt the dosage compensation complex to upregulate expression in a male-specific manner.

Supplemental Figure S10. The MRE motif is centered at roX2 ChIRP-seq peaks (HAS). The best-matched MRE motif within each roX2 ChIRP-seq peak (HAS) was calculated by CentriMo (Bailey et al. 2009) and plotted. In each species, the MRE motif is significantly centered, indicating the precision and high-fidelity with which ChIRP-seq can map precise roX binding sites.

Supplemental Figure S11. HAS on *D. willistoni* ME-D are similar to HAS on ME-A, despite its ancestry as an autosome.

(A) The MRE motifs from ME-A and ME-D are indistinguishable. We did not find evidence of tamed transposable elements at HAS on ME-D, as found on the more recently evolved neo-sex chromosome of the *D. miranda* (Ellison and Bachtrog 2013).

(B) ME-A and ME-D HAS are similarly distributed on genomic regions, and are enriched especially in introns. Note that UTRs are grouped with intergenic regions here.

(C) Intronic HAS are significantly biased towards the reverse-complement orientation of the MRE motif (CT-repeat) on both ME-A and ME-D.

(D) HAS are significantly proximal to PPT on both ME-A and ME-D.

Supplemental Figure S12. Overlap and proximity between homologous roX binding sites.

(A) Species-to-species liftover and HAS distance calculation strategy. Homologous regions in two species' genomes were mapped by genome-wide liftover. If homologous sites are both HAS, the HAS are overlapping and the distance between is 0. If a homologous site is a HAS in one species and not another, the distance to the nearest HAS is calculated (d>0). If the HAS has no nearby neighbor in the other species, the distance is much larger.

(B) Overlap and proximity between homologous HAS using the above strategy for all pairwise species comparisons. Though exact conservation of binding sites (i.e. distance = 0) between any two species is limited (approximately 10-30%), this is significantly higher than expected by random chance, as a random permutation of all HAS over their respective chromosomes or the whole genome yields very few overlapping peaks. Additionally, if exact peak overlap is lost (i.e. d>0), there is a high likelihood that another peak is nearby in the homologous genomic region, as indicated by the steep slope of the "observed" line in this distance regime. A higher percentage of peaks overlap in D.mel–D.wil and D.vir–D.bus than in other pairwise comparisons, perhaps reflecting the closer phylogenetic relationships of these pairs.

Supplemental Figure S13. Analyses of HAS in *D. willistoni* and *D. virilis* (supplementary to *D. melanogaster* analyses in **Fig. 6**).

(A) HAS are distributed over genomic regions (intergenic + UTRs, CDS, and introns), with particular enrichment in introns. Intergenic and UTRs are grouped together because UTRs are not reliably mapped in these species.

(B) HAS are significantly proximal to PPT in both *D. willistoni* and *D. virilis*.

(C) HAS are enriched in short introns in both *D. willistoni* and *D. virilis*, relative to all introns.

(D) Intronic HAS are significantly biased towards the reverse-complement orientation of the MRE motif (CT-repeat) in both *D. willistoni* and *D. virilis*. There is a weak bias towards the GA-repeat MRE motif in CDS in *D. willistoni*. Note that peak classifications differ from those in **Fig. 6**.

(E) The difference between the observed and random HAS (conserved, strong only) distributions on the X-chromosomes of *D. willistoni* and *D. virilis*. The positive *y*-value near the theoretically perfect spacing distance indicates an enrichment of the even spacing model relative to random spacing; conversely, the negative y-value at short distances indicates a depletion of the clustered spacing model relative to random spacing. This trend is not as robust in *D. willistoni*.

Supplemental Figure S14. Analysis of intronic and exonic HAS in *D. melanogaster*.

(A) HAS are significantly proximal to PPT relative to a random distribution. Approximately 20% of HAS are ±100bp from a PPT, vs. 7% in the random distribution.

(B) HAS-bearing introns are more pyrimidine-rich than typical introns.

(C) HAS-bearing introns are shorter than typical introns.

(D) HAS-bearing exons are more purine-rich than typical exons.

(E) HAS-bearing exons are slightly longer than typical exons.

Supplemental Figure S15. MRE can evolve from the polypyrimidine tract of introns.

(A) Genome browser tracks of roX2 ChIRP-seq at the *CG8097* locus in four species. In *D. melanogaster* and *D. willistoni*, there is a HAS in the first intron of *CG8097* (highlighted in gray), but this HAS is lost in *D. virilis* and *D. busckii*. In *D. virilis*, a new HAS is present in the putative 3'UTR of neighboring gene *CG9220*, illustrating the principle of HAS turnover in proximity.

(B) The highlighted sequence of the first intron of *CG8097* in four species (coding exons shown as black blocks). Instances of the MRE motif (red) are present within the pyrimidine-rich PPT (cyan box, approximately) of *CG8097*'s intron in *D. melanogaster* and *D. willistoni.* However, high-scoring incidences of the MRE motif are absent from the PPT of *CG8097*'s intron in *D. virilis* and *D. busckii*, in accord with the lack of a corresponding HAS in these two species. Thus, intronic PPT can serve as both an RNA signal (for splicing) and as a DCC binding site (MRE-bearing HAS).

(C) Genome browser tracks of roX ChIRP-seq at the *Ns3* locus in four species. In *D. melanogaster*, there is a HAS in the second intron of *Ns3* (highlighted in gray), but this HAS is absent in *D. willistoni*, *D. virilis,* and *D. busckii*. Most parsimoniously, this suggests that the HAS evolved in the *melanogaster* lineage. In *D. virilis*, a new HAS is present nearby, illustrating the principle of HAS turnover in proximity.

(D) The highlighted sequence of the second intron of *Ns3* in four species. Again, the MRE motif is present within the PPT of *Ns3's* intron in *D. melanogaster* and no high-scoring MRE motifs are found in *D. willistoni*, *D. virilis,* and *D. busckii*, in accord with the lack of a corresponding HAS in these three species.

Quinn_Fig.S1

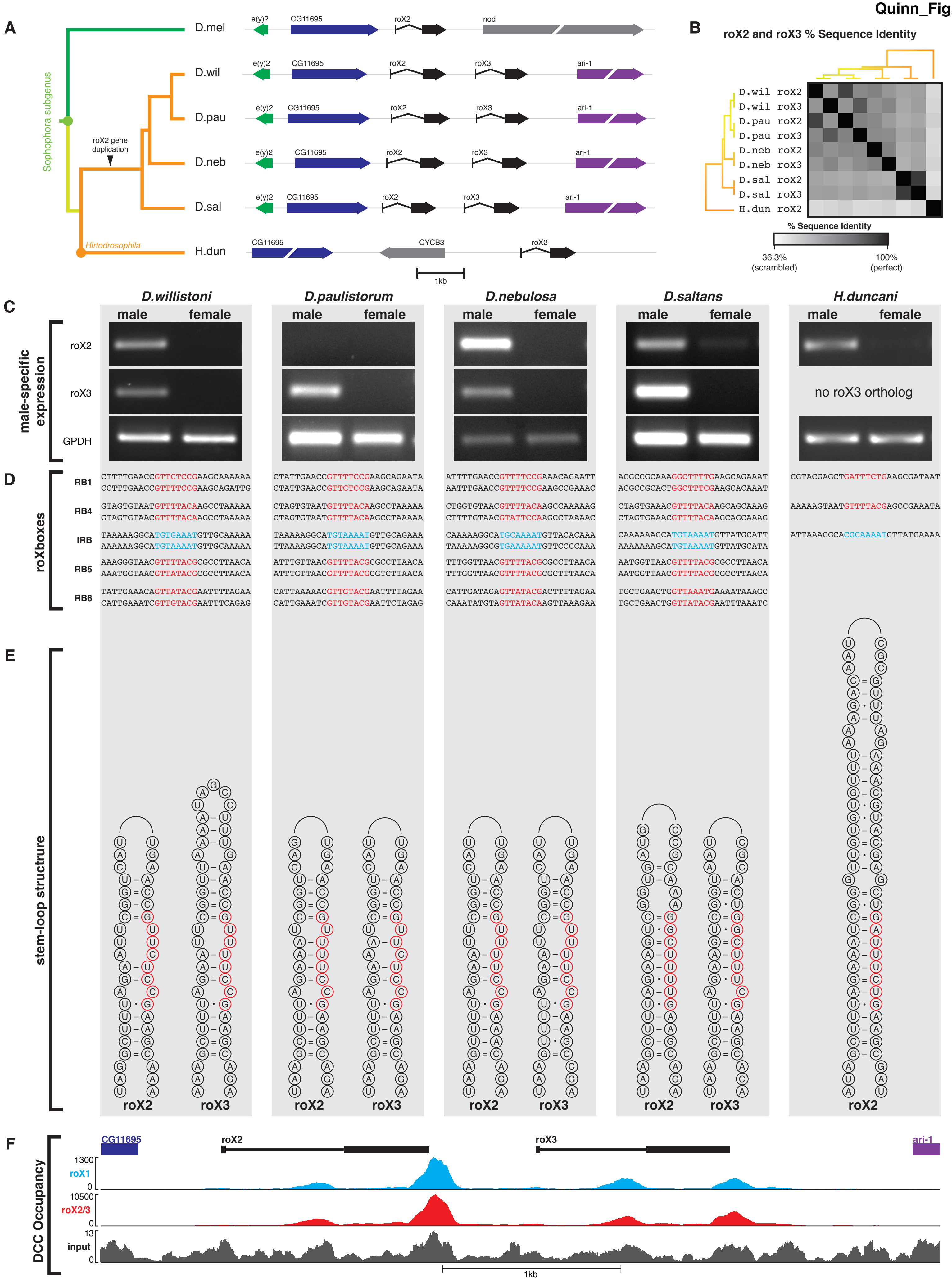
A
Sophophora subgenus
Hirtodrosophila
roX2 gene duplication
D.mel
D.wil
D.pau
D.neb
D.sal
H.dun
e(y)2
CG11695
roX2
roX3
nod
ari-1
CYCB3
1kb
B
roX2 and roX3 % Sequence Identity
D.wil roX2
D.wil roX3
D.pau roX2
D.pau roX3
D.neb roX2
D.neb roX3
D.sal roX2
D.sal roX3
H.dun roX2
% Sequence Identity
36.3% (scrambled)
100% (perfect)
C
male-specific expression
D.willistoni
D.paulistorum
D.nebulosa
D.saltans
H.duncani
male
female
roX2
roX3
GPDH
no roX3 ortholog
D
roXboxes
RB1
RB4
IRB
RB5
RB6
CTTTTGAACCGTTCTCCGAAGCAAAAAA
CCTTTGAACCGTTTTCCGAAGCAAGATTG
GTAGTGTAATGTTTTACAAGCCTAAAAA
GTAGTGTAACGTTTTACAAGCCTAAAAA
TAAAAAGGCATGTGAAATGTTGCAAAAA
AAAAAAGGCATGTAAAATGTTGCAAAAA
AAAGGGTAACGTTTTACGCGCCTTAACA
AAATGGTAACGTTATACGCGCCTTAACA
TATTGAAACAGTTATACGAATTTTAGAA
CATTGAAATCGTTGTACGAATTTCAGAG
CTATTGAACCGTTTTCCGAAGCAGAATA
CTATTGAACCGTTCTCCGAAGCAGAATA
CTAGTGTAATGTTTTACAAGCCTAAAAA
CTAGTGTAATGTTTTACAAGCCTAAAAA
TAAAAAGGCATGTAAAATGTTGCAGAAA
TTAAAAGGCATGTAAAATGTTGCAGAAA
ATTTGTTAACGTTTTACGCGCCTTAACA
ATTTGTTAACGTTTTACGCGTCTTAACA
CATTAAAAACGTTGTACGAATTTTAGAA
CATTGAAATCGTTGTACGAATTCTAGAG
ATTTTGAACCGTTTTCCGAAACAGAATT
AATTTGAACCGTTTTCCGAAGCCGAAAC
CTGGTGTAACGTTTTACAAGCCAAAAAG
CTTTTGTAACGTATTCCAAGCCTAAAAC
CAAAAAGGCATGCAAAATGTTACACAAA
TAAAAAGGCGTGAAAAATGTTCCCCAAA
TTTGGTTAACGTTTTACGCGCCTTAACA
TTTGGTTAACGTTTTACGCGCTTTAACA
CATTGATAGAGTTATACGACTTTTAGAA
CAAATATGTAGTTATACAAGTTAAAGAA
ACGCCGCAAAGGCTTTTGAAGCAGAAAT
ACGCCGCACTGGCTTTCGAAGCACAAAT
CTAGTGAAACGTTTTACAAGCAGCAAAG
CTAGTGAAACGTTTTACAAGCAGCAAAG
CAAAAAAGCATGTAAAATGTTATGCATT
AAAAAAAGCATGTAAAATGTTATGCATA
AATGGTTAACGTTTTACGCGCCTTAACA
AATGGTTAACGTTTTACGCGCCTTAACA
TGCTGAACTGGTTAAATGAAAATAAAGC
TGCTGAACTGGTTATACGAATTTAAATC
CGTACGAGCTGATTTCTGAAGCGATAAT
AAAAAGTAATGTTTTACGAGCCGAAATA
ATTAAAGGCACGCAAAATGTTATGAAAA
E
stem-loop structure
roX2
roX3
F
DCC Occupancy
CG11695
roX2
roX3
ari-1
1300
roX1
0
10500
roX2/3
0
13
input
0
1kb

Quinn_Fig.S2

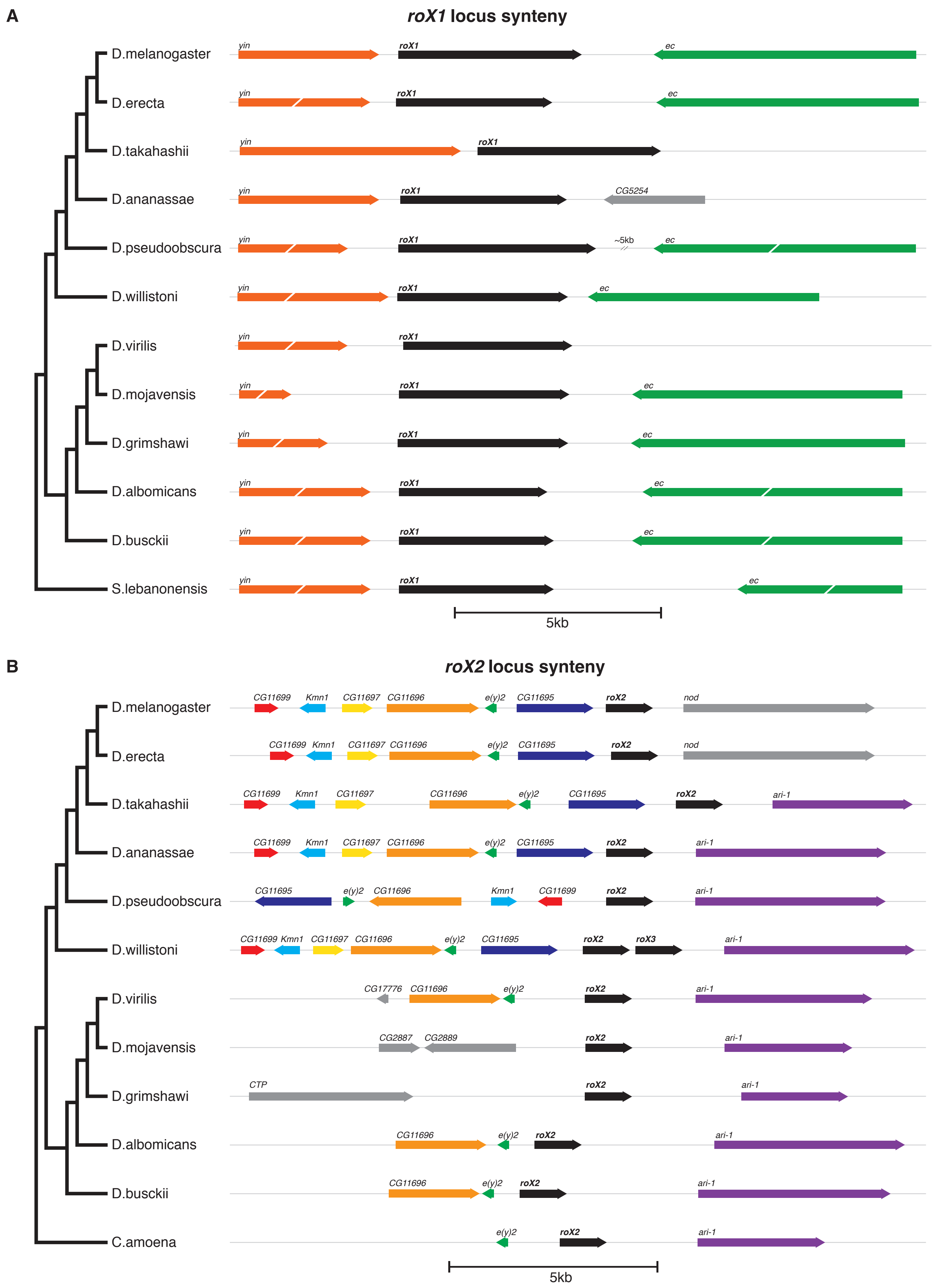
A
roX1 locus synteny
D.melanogaster
D.erecta
D.takahashii
D.ananassae
D.pseudoobscura
D.willistoni
D.virilis
D.mojavensis
D.grimshawi
D.albomicans
D.busckii
S.lebanonensis
yin
roX1
ec
CG5254
~5kb
5kb
B
roX2 locus synteny
D.melanogaster
D.erecta
D.takahashii
D.ananassae
D.pseudoobscura
D.willistoni
D.virilis
D.mojavensis
D.grimshawi
D.albomicans
D.busckii
C.amoena
CG11699
Kmn1
CG11697
CG11696
e(y)2
CG11695
roX2
roX3
nod
ari-1
CG17776
CG2887
CG2889
CTP
5kb

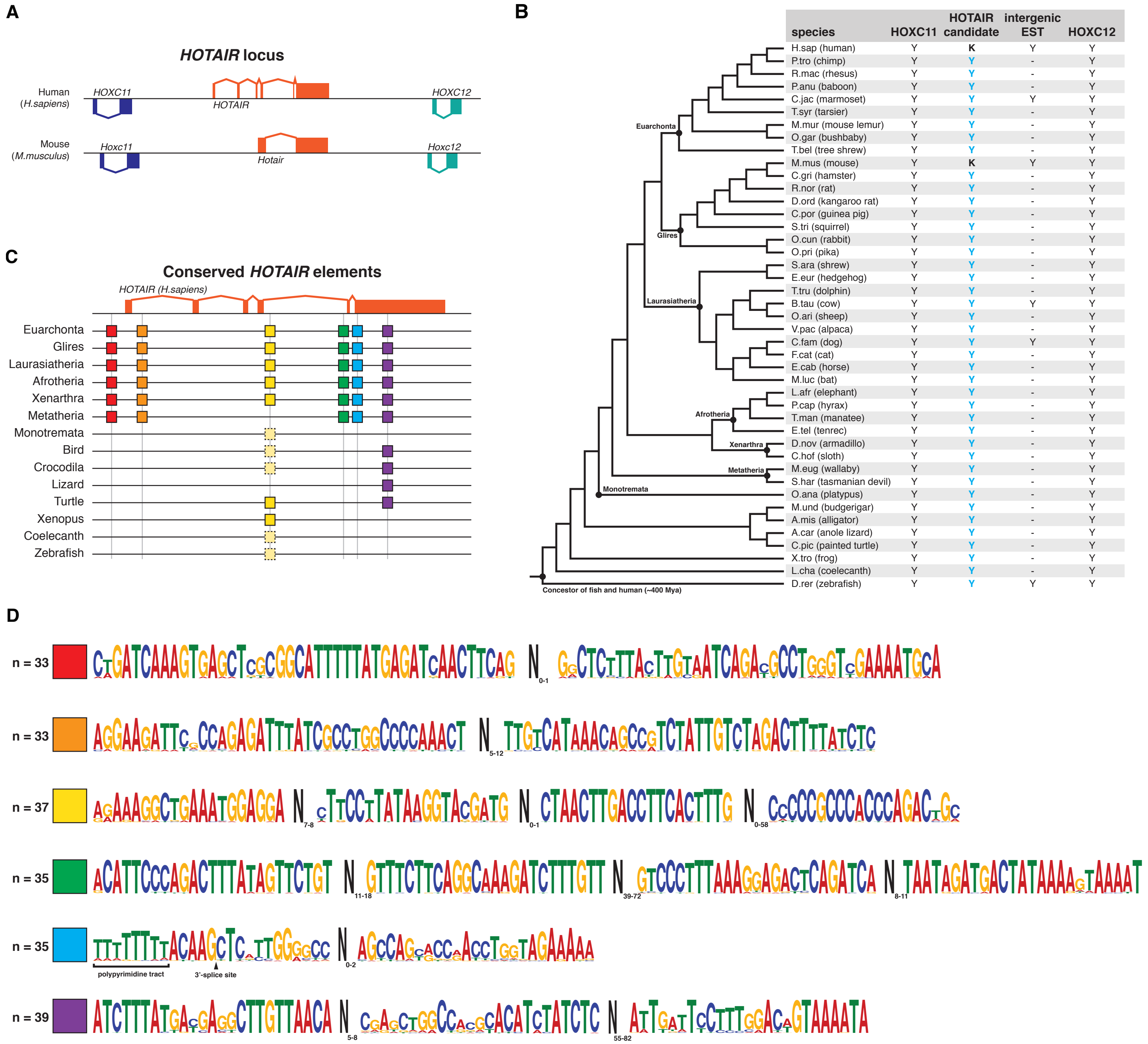
A
HOTAIR locus
Human (H.sapiens)
HOXC11
HOTAIR
HOXC12
Mouse (M.musculus)
Hoxc11
Hotair
Hoxc12
B
species | HOXC11 | HOTAIR candidate | intergenic EST | HOXC12
H.sap (human) | Y | K | Y | Y
P.tro (chimp) | Y | Y | - | Y
R.mac (rhesus) | Y | Y | - | Y
P.anu (baboon) | Y | Y | - | Y
C.jac (marmoset) | Y | Y | Y | Y
T.syr (tarsier) | Y | Y | - | Y
M.mur (mouse lemur) | Y | Y | - | Y
O.gar (bushbaby) | Y | Y | - | Y
T.bel (tree shrew) | Y | Y | - | Y
M.mus (mouse) | Y | K | Y | Y
C.gri (hamster) | Y | Y | - | Y
R.nor (rat) | Y | Y | - | Y
D.ord (kangaroo rat) | Y | Y | - | Y
C.por (guinea pig) | Y | Y | - | Y
S.tri (squirrel) | Y | Y | - | Y
O.cun (rabbit) | Y | Y | - | Y
O.pri (pika) | Y | Y | - | Y
S.ara (shrew) | Y | Y | - | Y
E.eur (hedgehog) | Y | Y | - | Y
T.tru (dolphin) | Y | Y | - | Y
B.tau (cow) | Y | Y | Y | Y
O.ari (sheep) | Y | Y | - | Y
V.pac (alpaca) | Y | Y | - | Y
C.fam (dog) | Y | Y | Y | Y
F.cat (cat) | Y | Y | - | Y
E.cab (horse) | Y | Y | - | Y
M.luc (bat) | Y | Y | - | Y
L.afr (elephant) | Y | Y | - | Y
P.cap (hyrax) | Y | Y | - | Y
T.man (manatee) | Y | Y | - | Y
E.tel (tenrec) | Y | Y | - | Y
D.nov (armadillo) | Y | Y | - | Y
C.hof (sloth) | Y | Y | - | Y
M.eug (wallaby) | Y | Y | - | Y
S.har (tasmanian devil) | Y | Y | - | Y
O.ana (platypus) | Y | Y | - | Y
M.und (budgerigar) | Y | Y | - | Y
A.mis (alligator) | Y | Y | - | Y
A.car (anole lizard) | Y | Y | - | Y
C.pic (painted turtle) | Y | Y | - | Y
X.tro (frog) | Y | Y | - | Y
L.cha (coelecanth) | Y | Y | - | Y
D.rer (zebrafish) | Y | Y | Y | Y
Euarchonta
Glires
Laurasiatheria
Afrotheria
Xenarthra
Metatheria
Monotremata
Concestor of fish and human (~400 Mya)
C
Conserved HOTAIR elements
HOTAIR (H.sapiens)
Euarchonta
Glires
Laurasiatheria
Afrotheria
Xenarthra
Metatheria
Monotremata
Bird
Crocodila
Lizard
Turtle
Xenopus
Coelecanth
Zebrafish
D
n = 33
n = 33
n = 37
n = 35
n = 35
n = 39
polypyrimidine tract
3'-splice site

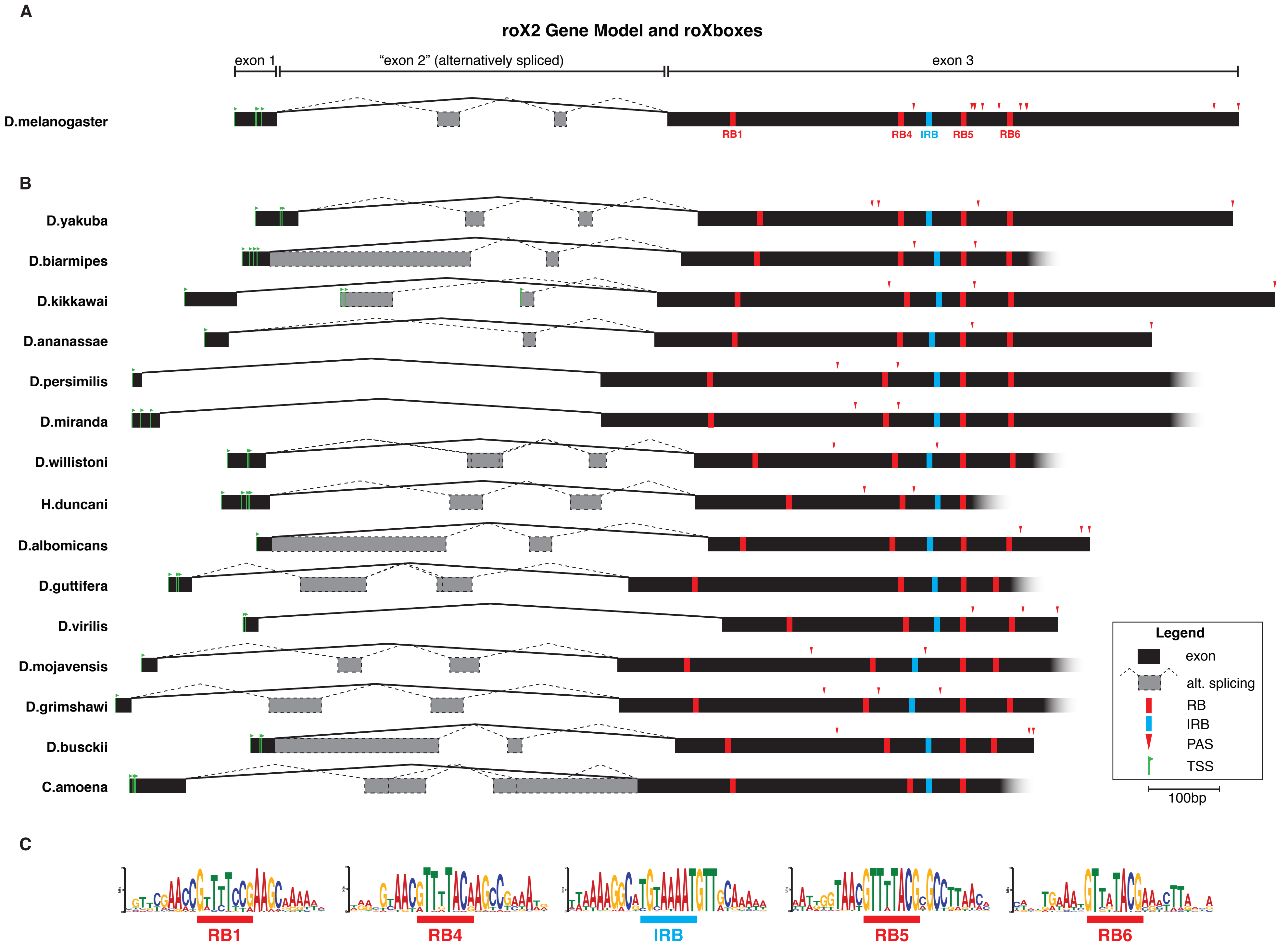
A
roX2 Gene Model and roXboxes
exon 1
"exon 2" (alternatively spliced)
exon 3
D.melanogaster
RB1
RB4
IRB
RB5
RB6
B
D.yakuba
D.biarmipes
D.kikkawai
D.ananassae
D.persimilis
D.miranda
D.willistoni
H.duncani
D.albomicans
D.guttifera
D.virilis
D.mojavensis
D.grimshawi
D.busckii
C.amoena
Legend
exon
alt. splicing
RB
IRB
PAS
TSS
100bp
C
RB1
RB4
IRB
RB5
RB6

Quinn_Fig.S5

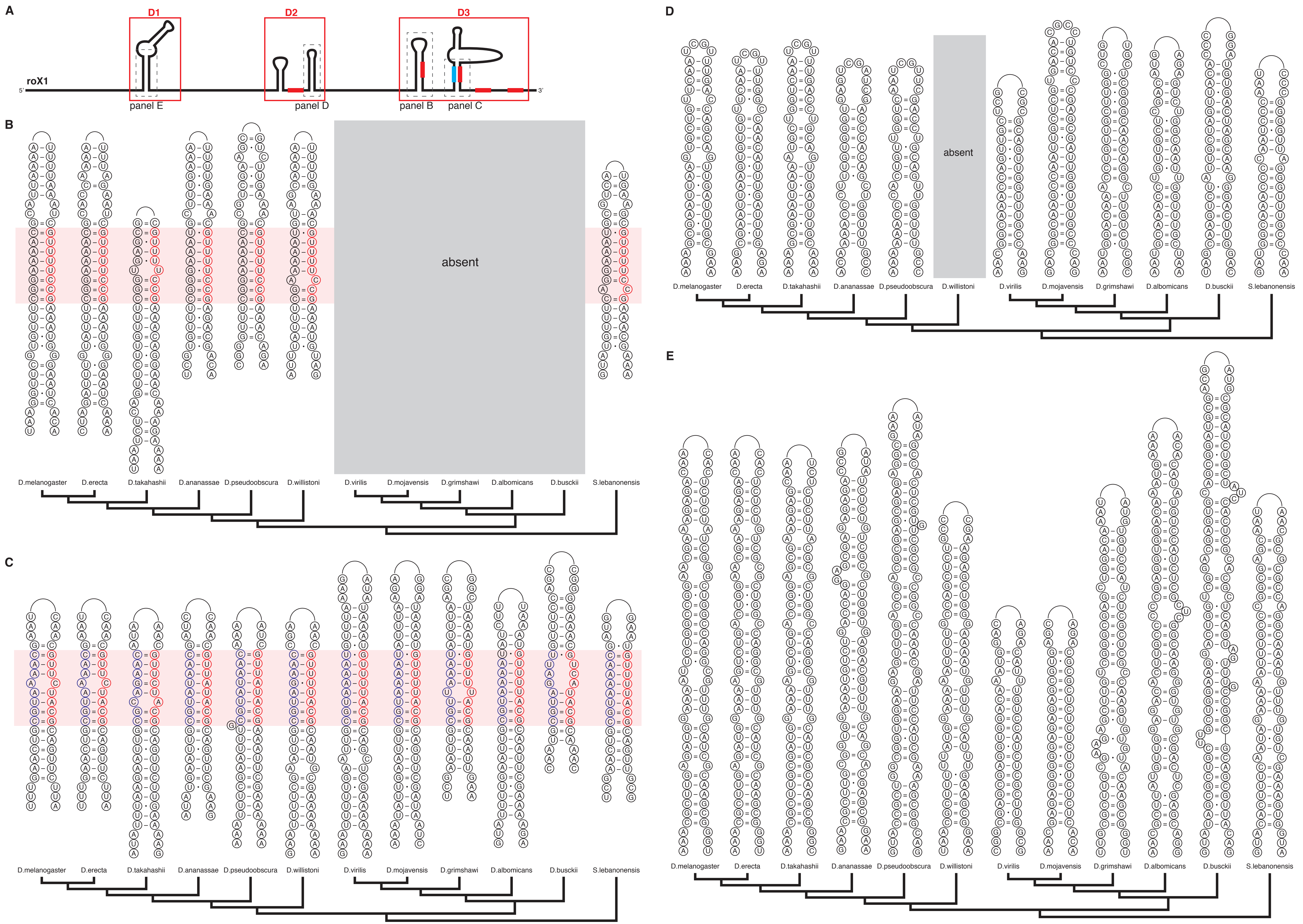
A
D1
D2
D3
roX1
5'
3'
panel E
panel D
panel B
panel C
B
absent
D.melanogaster
D.erecta
D.takahashii
D.ananassae
D.pseudoobscura
D.willistoni
D.virilis
D.mojavensis
D.grimshawi
D.albomicans
D.busckii
S.lebanonensis
C
D
E

Quinn_Fig.S6

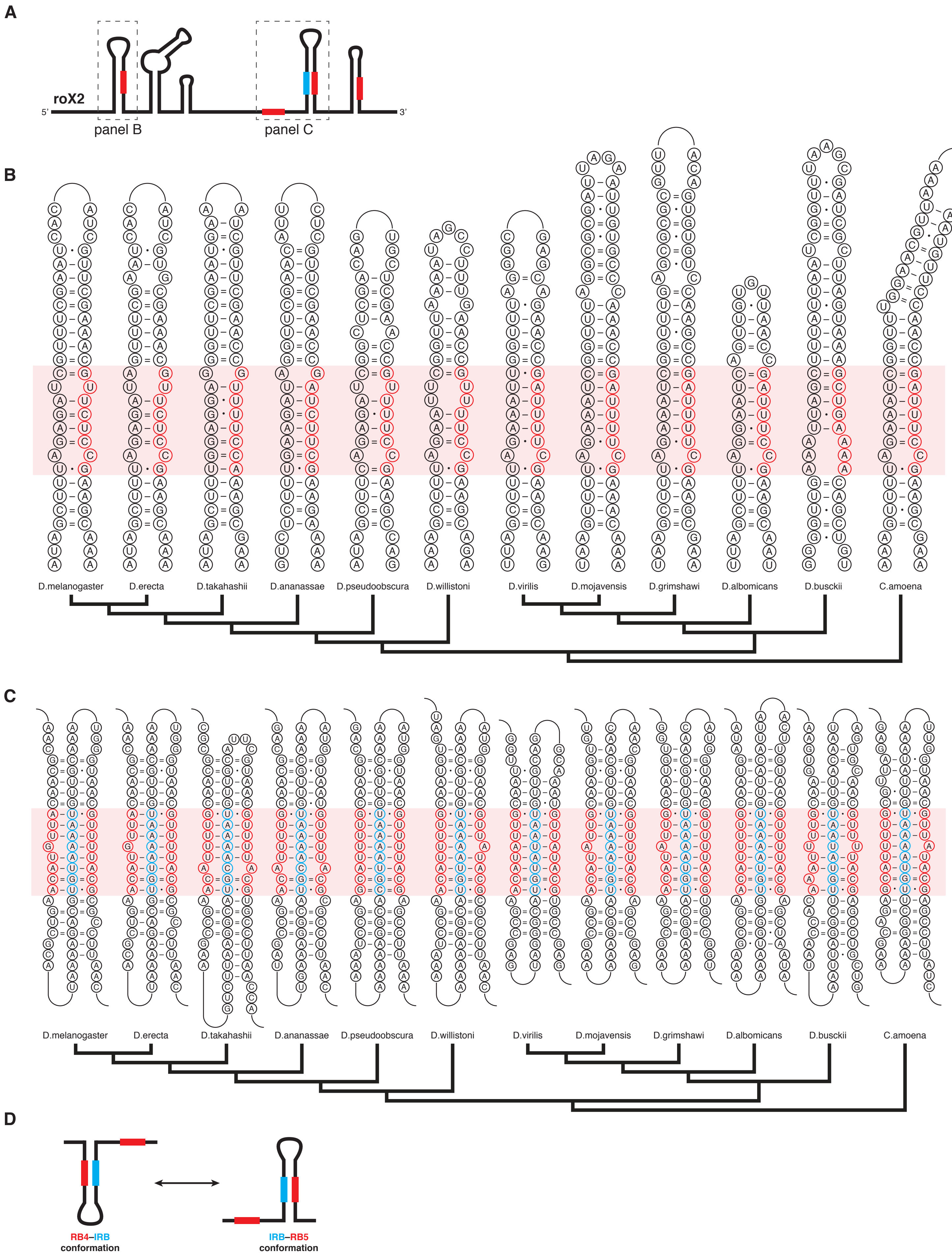
A
roX2
5'
3'
panel B
panel C
B
D.melanogaster
D.erecta
D.takahashii
D.ananassae
D.pseudoobscura
D.willistoni
D.virilis
D.mojavensis
D.grimshawi
D.albomicans
D.busckii
C.amoena
C
D.melanogaster
D.erecta
D.takahashii
D.ananassae
D.pseudoobscura
D.willistoni
D.virilis
D.mojavensis
D.grimshawi
D.albomicans
D.busckii
C.amoena
D
RB4–IRB
conformation
IRB–RB5
conformation

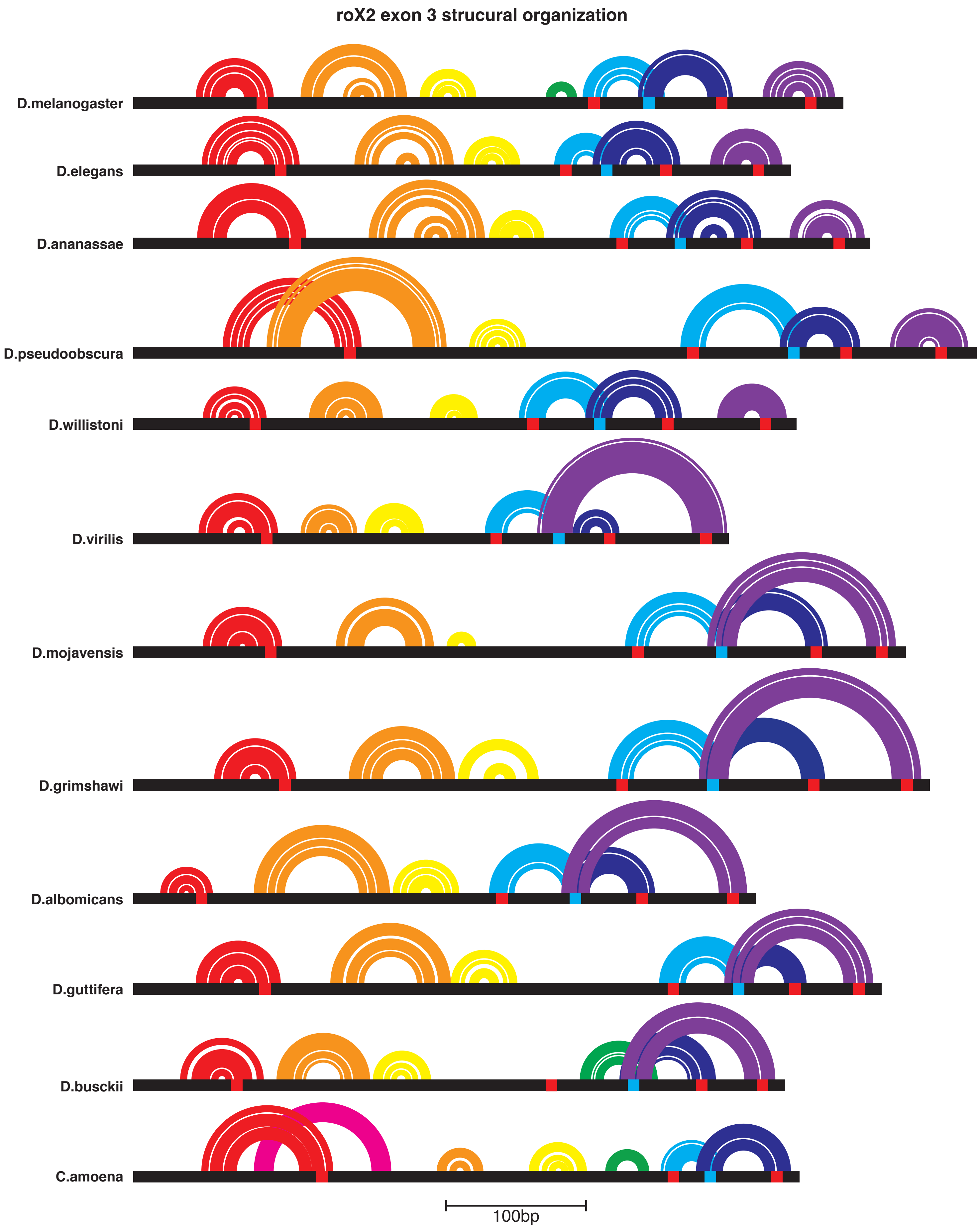
roX2 exon 3 strucural organization
D.melanogaster
D.elegans
D.ananassae
D.pseudoobscura
D.willistoni
D.virilis
D.mojavensis
D.grimshawi
D.albomicans
D.guttifera
D.busckii
C.amoena
100bp

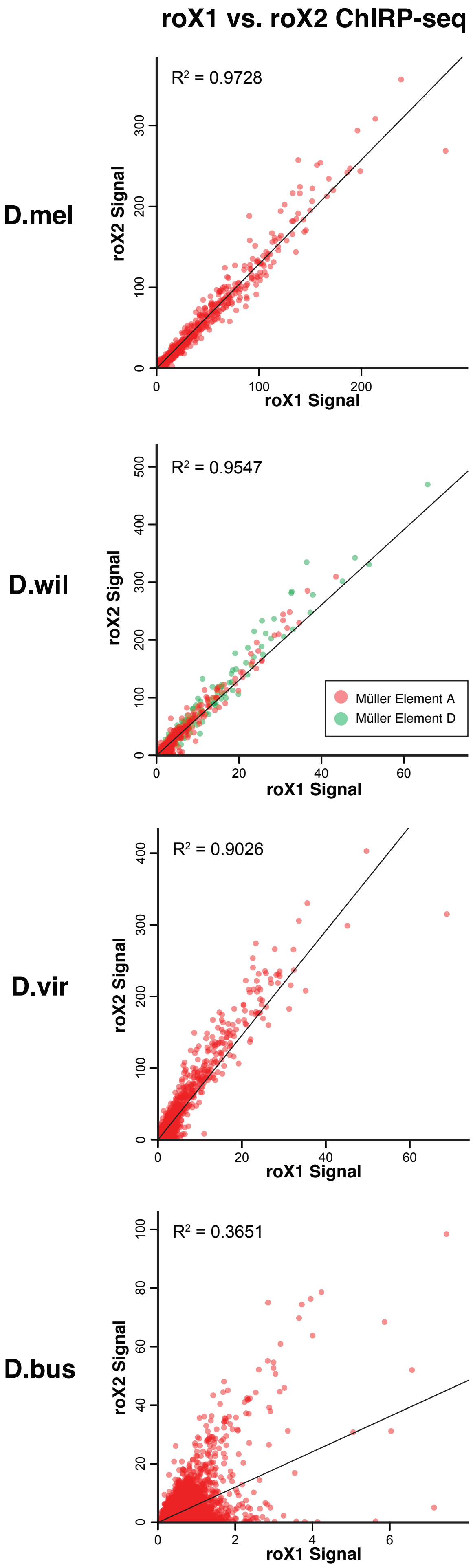
roX1 vs. roX2 ChIRP-seq
D.mel
R² = 0.9728
roX2 Signal
roX1 Signal
0
100
200
300
D.wil
R² = 0.9547
Müller Element A
Müller Element D
0
20
40
60
100
200
300
400
500
D.vir
R² = 0.9026
D.bus
R² = 0.3651
2
4
6
80

Quinn_Fig.S9

A

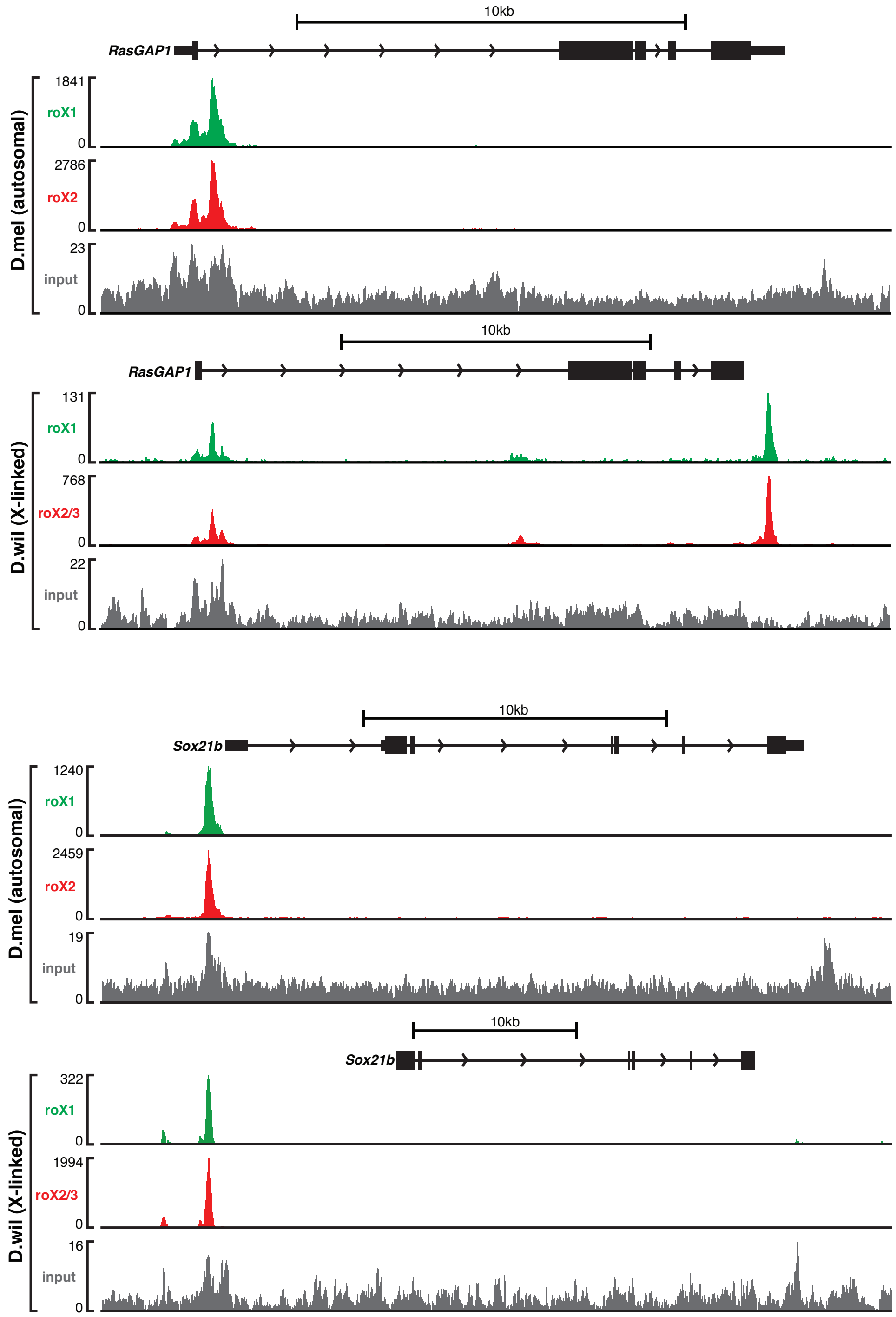

C

Autosomal HAS from roX ChIRP-seq (D.mel)

gene(s)	ME	region	fold-enrich
chinmo–CR45695	B	intergenic	101.9
CG34172	B	intron	40.8
bru-2	B	TSS	29.4
CR44856–CG42680	B	intergenic	54.9
dac	B	TSS	82.1
tup	B	TSS	29.4
df31	B	intron	78.4
CAP	C	TSS	28.7
psq	C	TSS	38.8
spin	C	intron	35.1
trh	D	TSS	21.8
Msr-110	D	TSS	48.9
Rac2	D	TSS	43.4
RasGap1	D	intron	97.1
RasGap1	D	TSS	45.8
tna	D	TSS	34.0
CR44714–CR44719	D	intergenic	29.3
CR43431	D	TSS	60.3
sox21b	D	TSS	98.9
pzg–CG12974	D	TSS	22.3
hth	E	TSS	24.0
mod(mdg4)	E	intron	40.7
InR	E	intron	41.1
fd96Cb	E	TSS	32.7
Ets98B	E	TSS	31.4
toy	F	TSS	29.8

Position of MRE Motif relative to roX ChIRP-seq Peak Summits (CentriMo)

D.mel

D.wil

D.vir

D.bus

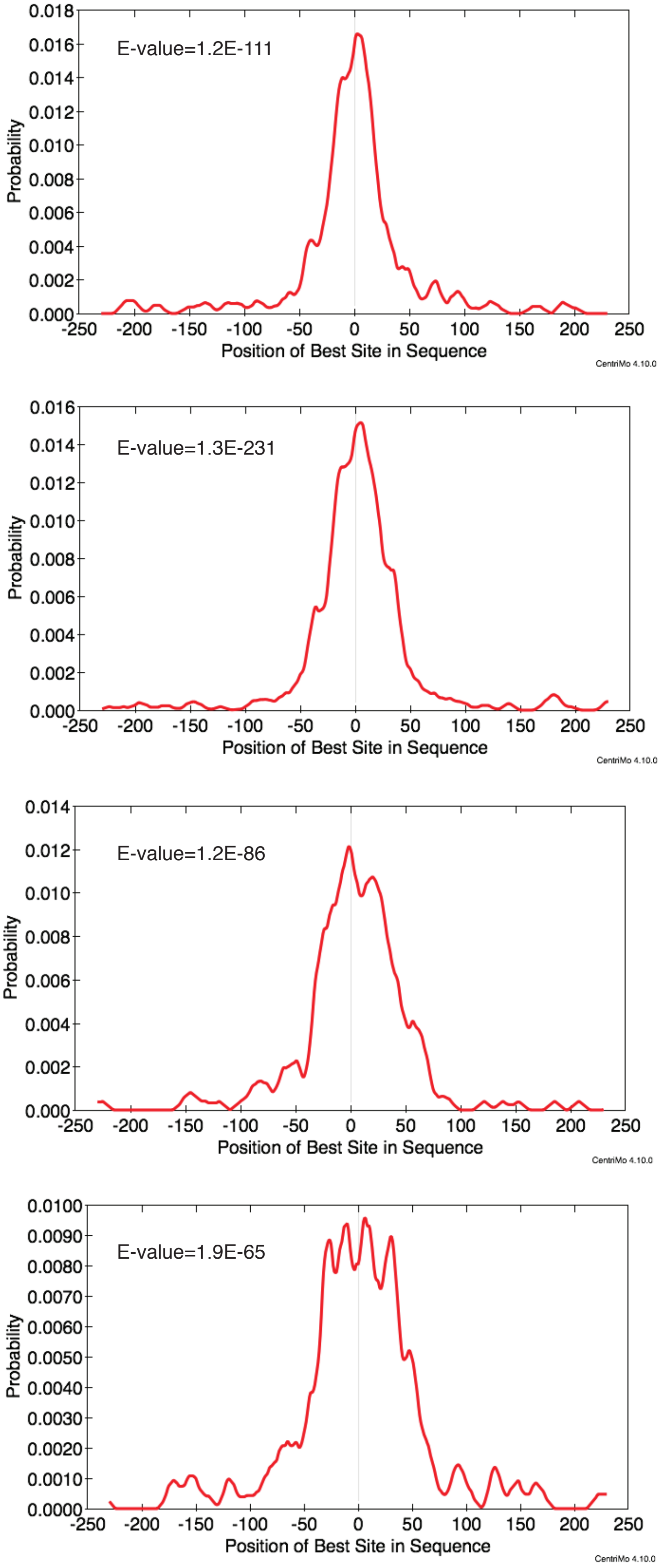

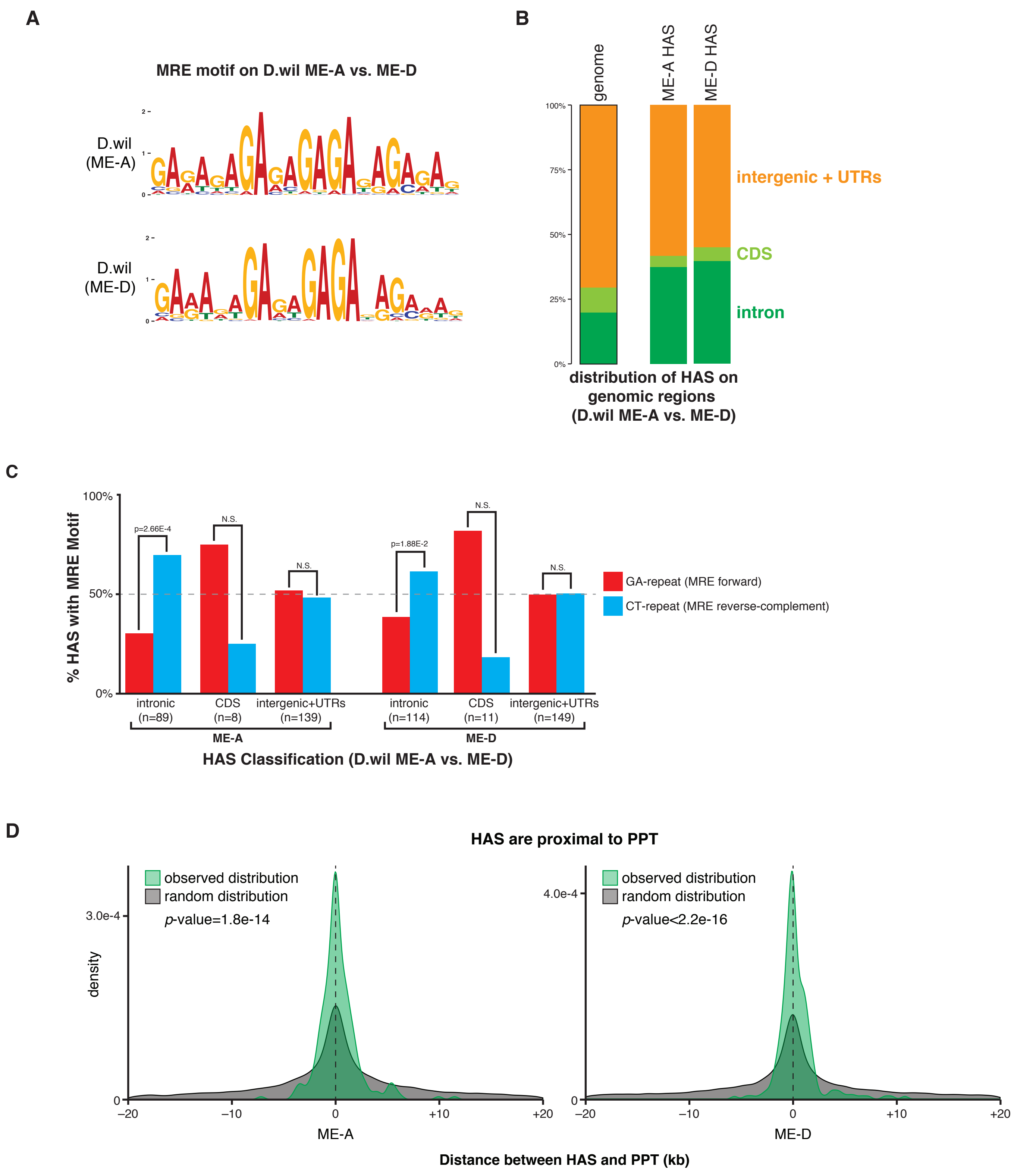
A
MRE motif on D.wil ME-A vs. ME-D
D.wil (ME-A)
D.wil (ME-D)
B
genome
ME-A HAS
ME-D HAS
100%
75%
50%
25%
0%
intergenic + UTRs
CDS
intron
distribution of HAS on genomic regions (D.wil ME-A vs. ME-D)
C
% HAS with MRE Motif
100%
50%
0%
p=2.66E-4
N.S.
N.S.
p=1.88E-2
N.S.
N.S.
GA-repeat (MRE forward)
CT-repeat (MRE reverse-complement)
intronic (n=89)
CDS (n=8)
intergenic+UTRs (n=139)
intronic (n=114)
CDS (n=11)
intergenic+UTRs (n=149)
ME-A
ME-D
HAS Classification (D.wil ME-A vs. ME-D)
D
HAS are proximal to PPT
observed distribution
random distribution
p-value=1.8e-14
p-value<2.2e-16
density
3.0e-4
4.0e-4
0
−20
−10
0
+10
+20
ME-A
ME-D
Distance between HAS and PPT (kb)

A

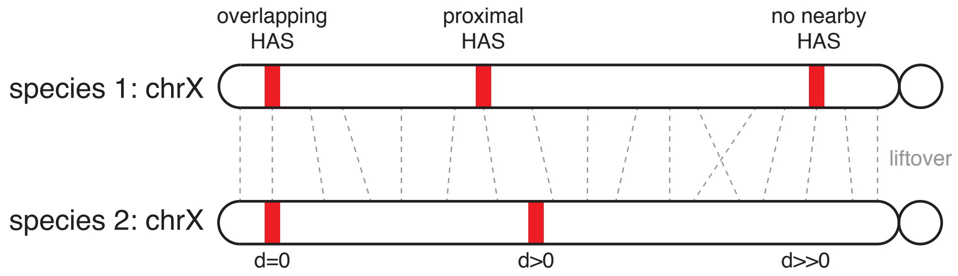
overlapping
HAS
proximal
HAS
no nearby
HAS
species 1: chrX
liftover
species 2: chrX
d=0
d>0
d>>0

B

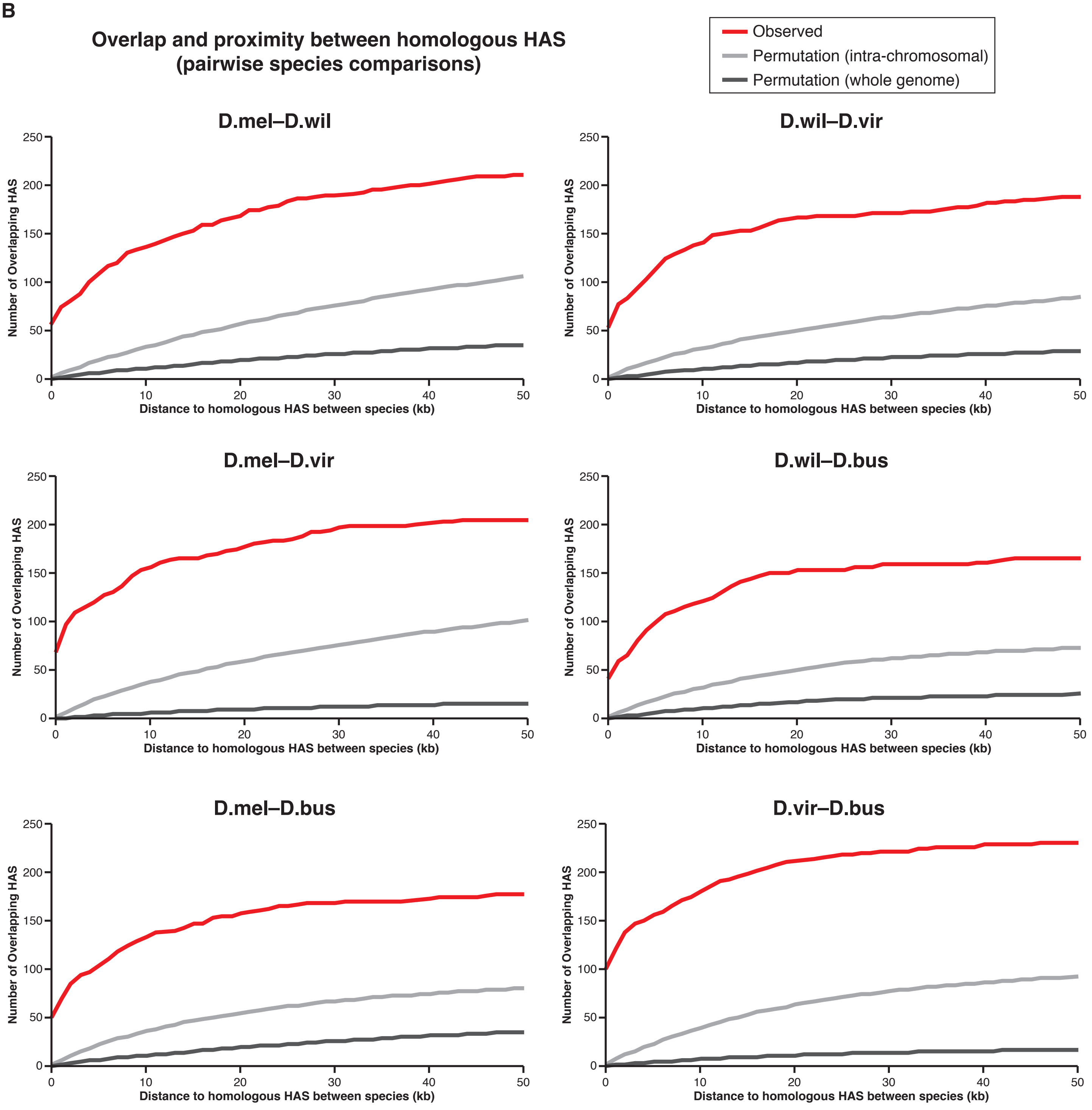
Overlap and proximity between homologous HAS
(pairwise species comparisons)
Observed
Permutation (intra-chromosomal)
Permutation (whole genome)
D.mel–D.wil
D.wil–D.vir
D.mel–D.vir
D.wil–D.bus
D.mel–D.bus
D.vir–D.bus
Number of Overlapping HAS
Distance to homologous HAS between species (kb)
0
50
100
150
200
250
0
10
20
30
40
50

Quinn_Fig.S13

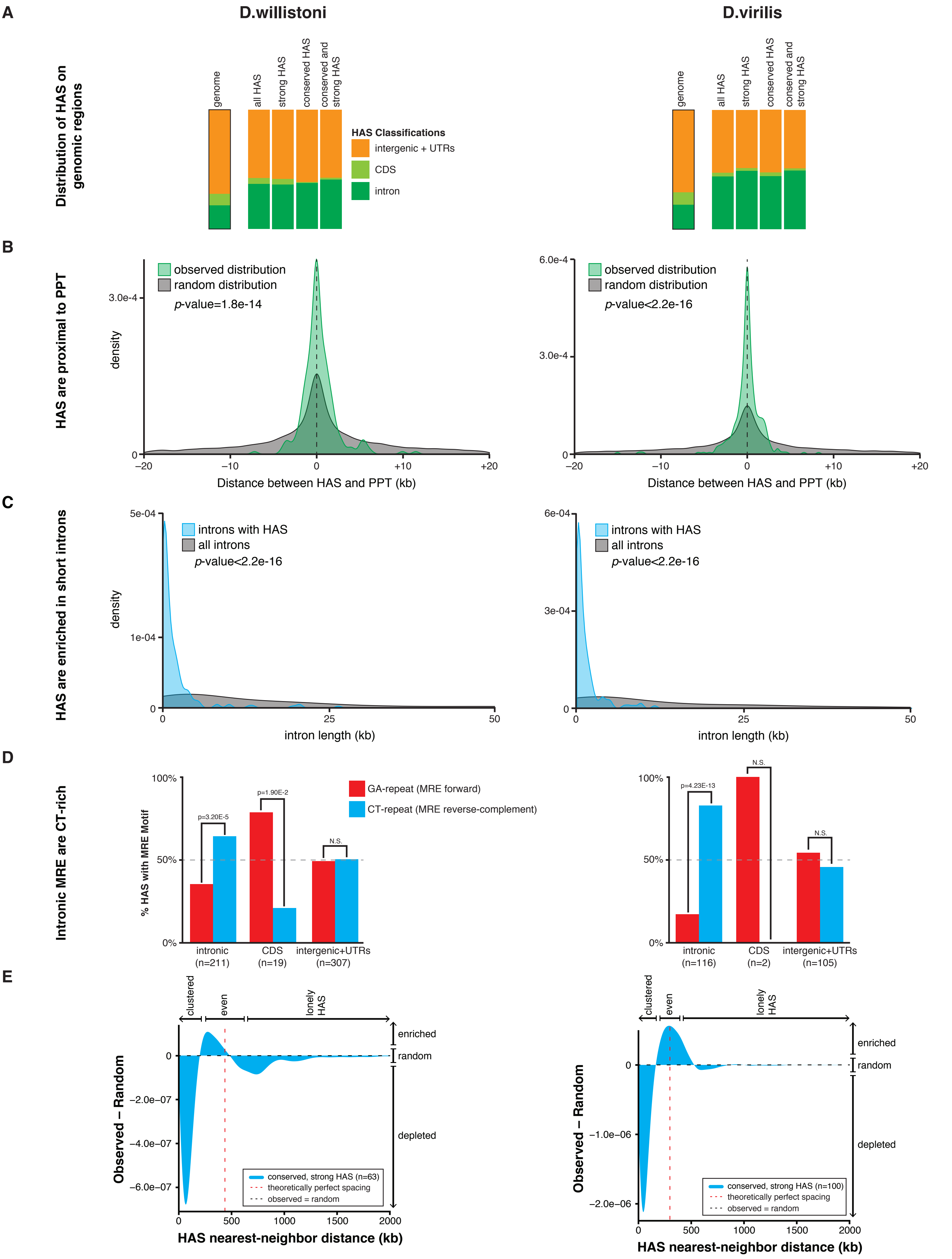

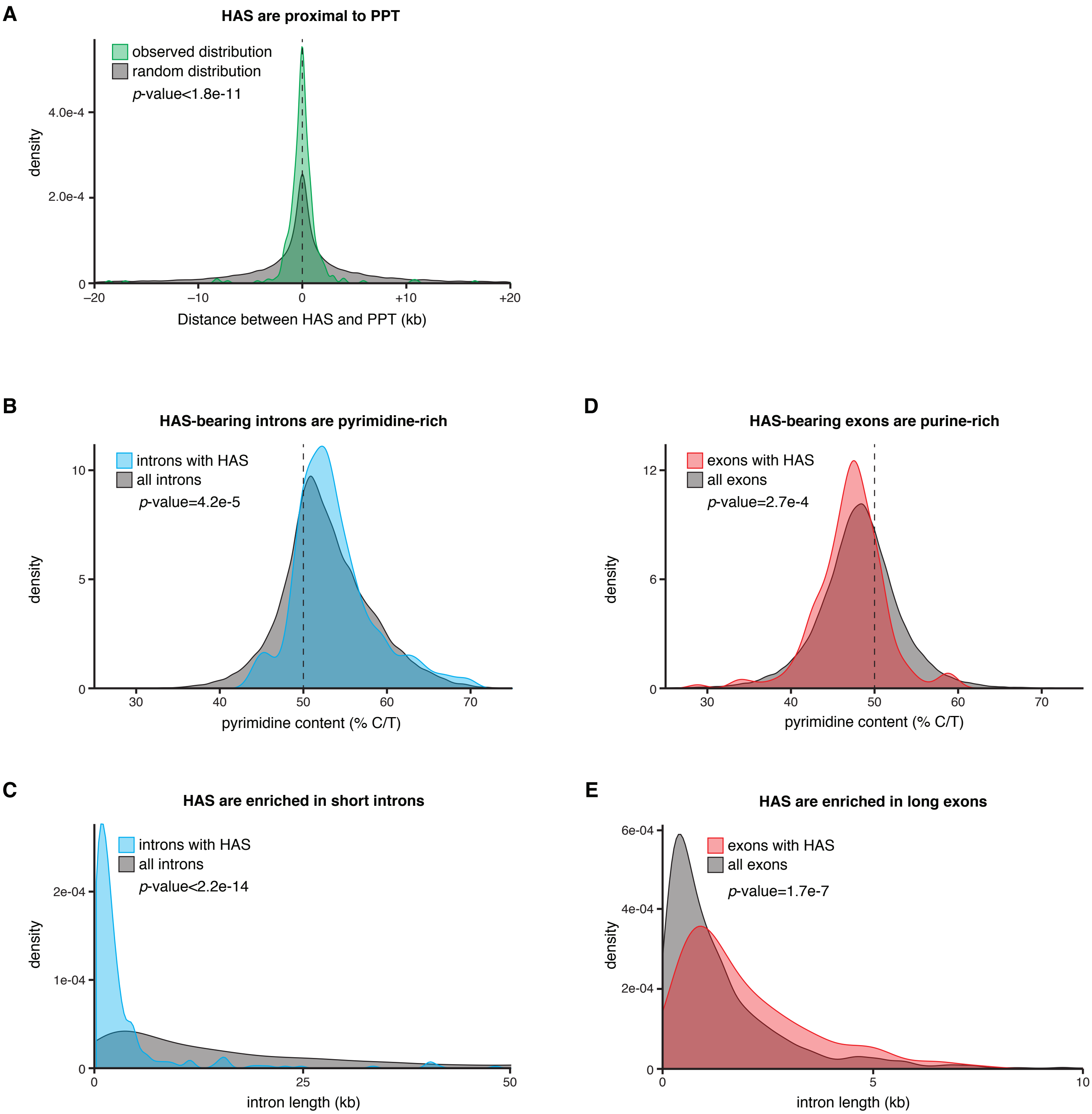
A
HAS are proximal to PPT
observed distribution
random distribution
p-value<1.8e-11
density
4.0e-4
2.0e-4
0
–20
–10
0
+10
+20
Distance between HAS and PPT (kb)
B
HAS-bearing introns are pyrimidine-rich
introns with HAS
all introns
p-value=4.2e-5
density
10
5
0
30
40
50
60
70
pyrimidine content (% C/T)
D
HAS-bearing exons are purine-rich
exons with HAS
all exons
p-value=2.7e-4
density
12
6
0
30
40
50
60
70
pyrimidine content (% C/T)
C
HAS are enriched in short introns
introns with HAS
all introns
p-value<2.2e-14
density
2e-04
1e-04
0
0
25
50
intron length (kb)
E
HAS are enriched in long exons
exons with HAS
all exons
p-value=1.7e-7
density
6e-04
4e-04
2e-04
0
0
5
10
intron length (kb)

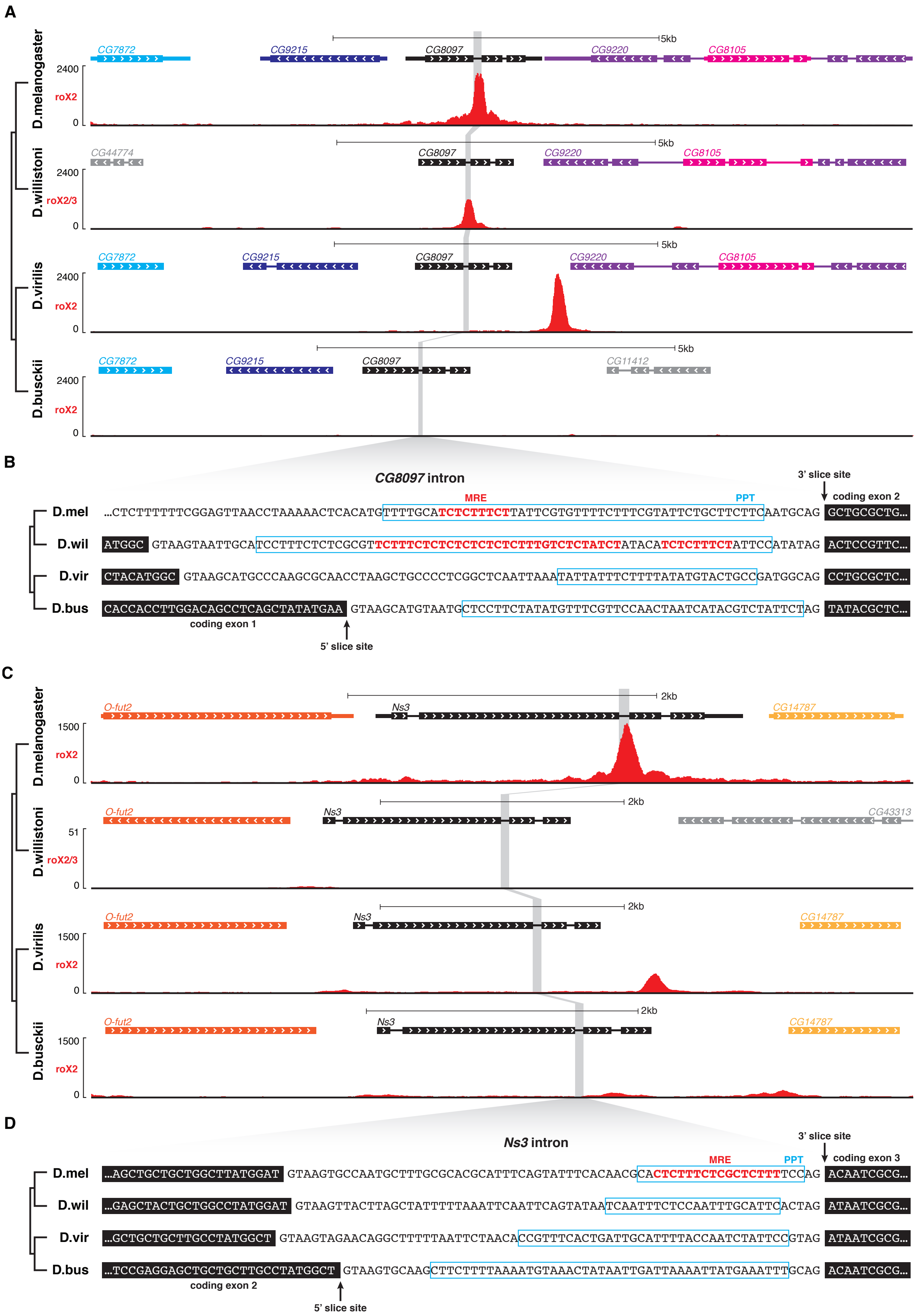
A
D.melanogaster
D.willistoni
D.virilis
D.busckii
roX2
roX2/3
2400
0
5kb
CG7872
CG9215
CG8097
CG9220
CG8105
CG44774
CG11412
B
CG8097 intron
3' slice site
coding exon 2
MRE
PPT
D.mel …CTCTTTTTTCGGAGTTAACCTAAAAACTCACATGTTTTGCATCTCTTTCTTATTCGTGTTTTCTTTCGTATTCTGCTTCTTCAATGCAG GCTGCGCTG…
D.wil ATGGC GTAAGTAATTGCATCCTTTCTCTCGCGTTCTTTCTCTCTCTCTCTCTTTGTCTCTATCTATACATCTCTTTCTATTCCATATAG ACTCCGTTC…
D.vir CTACATGGC GTAAGCATGCCCAAGCGCAACCTAAGCTGCCCCTCGGCTCAATTAAATATTATTTCTTTTATATGTACTGCCGATGGCAG CCTGCGCTC…
D.bus CACCACCTTGGACAGCCTCAGCTATATGAA GTAAGCATGTAATGCTCCTTCTATATGTTTCGTTCCAACTAATCATACGTCTATTCTAG TATACGCTC…
coding exon 1
5' slice site
C
1500
51
2kb
O-fut2
Ns3
CG14787
CG43313
D
Ns3 intron
3' slice site
coding exon 3
MRE
PPT
D.mel …AGCTGCTGCTGGCTTATGGAT GTAAGTGCCAATGCTTTGCGCACGCATTTCAGTATTTCACAACGCACTCTTTCTCGCTCTTTTCCAG ACAATCGCG…
D.wil …GAGCTACTGCTGGCCTATGGAT GTAAGTTACTTAGCTATTTTTAAATTCAATTCAGTATAATCAATTTCTCCAATTTGCATTCACTAG ATAATCGCG…
D.vir …GCTGCTGCTTGCCTATGGCT GTAAGTAGAACAGGCTTTTTAATTCTAACACCGTTTCACTGATTGCATTTTACCAATCTATTCCGTAG ATAATCGCG…
D.bus …TCCGAGGAGCTGCTGCTTGCCTATGGCT GTAAGTGCAAGCTTCTTTTAAAATGTAAACTATAATTGATTAAAATTATGAAATTTGCAG ACAATCGCG…
coding exon 2
5' slice site
